# Tools for assessing health research partnership outcomes and impacts: a systematic review

**DOI:** 10.1186/s12961-022-00937-9

**Published:** 2023-01-05

**Authors:** K. J. Mrklas, J. M. Boyd, S. Shergill, S. Merali, M. Khan, L. Nowell, A. Goertzen, L. M. Pfadenhauer, K. Paul, K. M. Sibley, L. Swain, M. Vis-Dunbar, M. D. Hill, S. Raffin-Bouchal, M. Tonelli, I. D. Graham

**Affiliations:** 1 Department of Community Health Sciences, Cumming School of Medicine, University of Calgary, 3D10, 3280 Hospital Drive NW, Calgary, AB T2N 4Z6 Canada; 2 Strategic Clinical Networks™, Provincial Clinical Excellence, Alberta Health Services, Calgary, AB Canada; 3 Knowledge Translation Program, St Michael’s Hospital, Li Ka Shing Knowledge Institute, Unity Health Toronto, Toronto, ON Canada; 4 Cumming School of Medicine, University of Calgary, Calgary, AB Canada; 5 Faculty of Kinesiology, University of Calgary, Calgary, AB Canada; 6 Department of Community Health Sciences, University of Manitoba, Winnipeg, MB Canada; 7 Faculty of Nursing, University of Calgary, Calgary, AB Canada; 8 Faculty of Science, University of Alberta, Edmonton, AB Canada; 9 Institute for Medical Information Processing, Biometry, and Epidemiology–IBE, Ludwig-Maximilian Universität Munich, Munich, Germany; 10 Pettenkofer School of Public Health, Munich, Germany; 11 University of Calgary Summer Studentships Program, Calgary, AB Canada; 12 George & Fay Yee Centre for Healthcare Innovation, University of Manitoba, Winnipeg, MB Canada; 13 University of British Columbia - Okanagan, Kelowna, BC Canada; 14 Departments of Clinical Neurosciences, Medicine and Radiology, Cumming School of Medicine, University of Calgary, Calgary, AB Canada; 15 Hotchkiss Brain Institute, Cumming School of Medicine, University of Calgary, Calgary, AB Canada; 16 Department of Medicine, Cumming School of Medicine, University of Calgary, Calgary, AB Canada; 17 Office of the Vice-President (Research), University of Calgary, Calgary, AB Canada; 18 Centre for Implementation Research, Ottawa Hospital Research Institute, Ottawa, ON Canada; 19 School of Epidemiology and Public Health & School of Nursing, University of Ottawa, Ottawa, ON Canada

**Keywords:** Health research partnerships, Evaluation tools, Psychometrics, Acceptability, Systematic review

## Abstract

**Objective:**

To identify and assess the globally available valid, reliable and acceptable tools for assessing health research partnership outcomes and impacts.

**Methods:**

We searched Ovid MEDLINE, Embase, CINAHL Plus and PsycINFO from origin to 2 June 2021, without limits, using an a priori strategy and registered protocol. We screened citations independently and in duplicate, resolving discrepancies by consensus and retaining studies involving health research partnerships, the development, use and/or assessment of tools to evaluate partnership outcomes and impacts, and reporting empirical psychometric evidence. Study, tool, psychometric and pragmatic characteristics were abstracted using a hybrid approach, then synthesized using descriptive statistics and thematic analysis. Study quality was assessed using the quality of survey studies in psychology (Q-SSP) checklist.

**Results:**

From 56 123 total citations, we screened 36 027 citations, assessed 2784 full-text papers, abstracted data from 48 studies and one companion report, and identified 58 tools. Most tools comprised surveys, questionnaires and scales. Studies used cross-sectional or mixed-method/embedded survey designs and employed quantitative and mixed methods. Both studies and tools were conceptually well grounded, focusing mainly on outcomes, then process, and less frequently on impact measurement. Multiple forms of empirical validity and reliability evidence was present for most tools; however, psychometric characteristics were inconsistently assessed and reported. We identified a subset of studies (22) and accompanying tools distinguished by their empirical psychometric, pragmatic and study quality characteristics. While our review demonstrated psychometric and pragmatic improvements over previous reviews, challenges related to health research partnership assessment and the nascency of partnership science persist.

**Conclusion:**

This systematic review identified multiple tools demonstrating empirical psychometric evidence, pragmatic strength and moderate study quality. Increased attention to psychometric and pragmatic requirements in tool development, testing and reporting is key to advancing health research partnership assessment and partnership science.

PROSPERO CRD42021137932

**Supplementary Information:**

The online version contains supplementary material available at 10.1186/s12961-022-00937-9.

## Scoping review and Coordinated Multicentre Team Protocol registrations

1. PROSPERO Protocol Registration: CRD42021137932 https://www.crd.york.ac.uk/prospero/display_record.php?RecordID=137932
2. Open Science Framework (Coordinated Multicentre Team Protocol): https://osf.io/gvr7y/
3. Coordinated Multicentre Team Protocol publication: https://systematicreviewsjournal.biomedcentral.com/articles/10.1186/s13643-018-0879-2

## Background

The emphasis on and number of studies involving health research partnerships has grown substantially over the last decade [1]. Despite this evolving popularity and mounting demand for the systematic quantification of partnership outcomes and impacts, the assessment of health research partnerships has not kept pace [2]. Here, we refer to health research partnerships as those involving “individuals, groups or organizations engaged in collaborative, health research activity involving at least one researcher (e.g. individual affiliated with an academic department, hospital or medical centre), and any partner actively engaged in any part of the research process (e.g. decision or policy maker, healthcare administrator or leader, community agency, charities, network, patients, industry partner, etc.)”(p 4) [3]﻿.

Although quantitative tools for assessing the outcomes and impacts of health research partnerships emerged in the late 1980s to early 1990s [5–7], available tools are largely simplistic and the assessment of outcomes and impacts in the health research partnerships domain, nascent [5, 7–13]. Available studies are often hampered by a lack of rigorous measurement, including tool psychometric testing to establish evidence of validity and reliability. The limitations of existing studies fall into three categories: many primary studies select single-use and locally relevant tools as a core part of the partnership process, with a focus on monitoring their partnerships’ progress and on bespoke outcomes and impacts of highest relevance to them [5, 9]. Although most tool studies aim to incorporate partner views, track individual partnership progression and capture partner perspectives, few aim to create more universally applicable, standardized tools that can be used more broadly or for replication studies [10]. Second, many such studies are limited by small sample sizes and lack of iterative tool testing, which in turn contributes to the lack of psychometric evidence and a lack of evidence across a broader range of contexts. Third, primary studies in this domain are often limited by interchanging terminology, a lack of discrete concept definitions, problems associated with literature indexing, location and retrieval [3, 14, 15], and multiple tool-specific challenges including construct identification, definition, refinement and application [5–10, 12].

Cumulatively, these challenges inhibit the evolution of partnership assessment and ultimately slow the advancement of partnership science [9, 10]. A recent overview of reviews examining quantitative measures to evaluate impact in research coproduction suggests that investigators must “engage more openly and critically with psychometric *and* pragmatic considerations when designing, implementing, [evaluating] and reporting on measurement tools” (p. 163) [8]. There is an established rationale for developing robust, pragmatic measures that are both relevant to partners and usable in real-world settings; pragmatic tools are viewed as a critical accompaniment to pragmatic designs [16–18]. In this light, health research partnership tools should be relevant to partners, be actionable, have a low completion burden, and demonstrate adequate validity and reliability. Importantly, there is a need for tools that are broadly applicable, can be used for benchmarks with accompanying norms to aid interpretation, and that demonstrate strong psychometric and theoretical underpinnings, without causing harm [16]. Closing these gaps would help to facilitate tool use, advance the measurement of systematic partnerships and drive improvements in partnership science [8].

Numerous tools for assessing health partnership outcomes and impacts have been identified in previous reviews focused on specific partnership domains, partner groups or contexts [5–12]; however, scope restrictions in these reviews preclude our understanding of tools *across* health research partnership traditions. These reviews also reveal that information about tool psychometric and pragmatic properties remains lacking. This study reviewed and systematically assessed globally available tools for the assessment of health research partnership outcomes and impacts to address documented gaps in both the psychometric and pragmatic characteristics of these assessment tools.

Our primary research question was as follows: what are the globally available, valid, reliable and acceptable tools for assessing the outcomes and impacts of health research partnerships? Our secondary research questions pertained to tool characteristics, including the following: what are the reported purposes of the tools, are outcomes and/or impacts measured, and what are the reported theoretical underpinnings and psychometric and pragmatic properties of the tools? (Additional file 1: Appendix S1). Secondary research questions pertaining to partnership characteristics were captured and will be reported in a forthcoming publication to preserve manuscript clarity.

## Methods

This review is part of a comprehensive, multisite synthesis effort by the Integrated Knowledge Translation Research Network (IKTRN) [3, 19] and was guided by a collaboratively built conceptual framework [3]. In this review, we define tools as “instruments (e.g. survey, measures, assessments, inventory, checklist, questionnaires, list of factors, subscales or similar) that can be used to assess the outcome or impact elements or domains of a health research partnership” (p 5)[3, 20].

The overall approach to the review was guided by the steps outlined by Arksey and O’Malley [21], with refinements [22–24], and additional guidance from the Centre for Reviews and Dissemination (CRD) guidance for undertaking reviews in healthcare [25], the Cochrane Handbook for Systematic Reviews [26] and the Joanna Briggs Institute Reviewers’ Manual [27]. This manuscript was structured and reported using the newly updated Preferred Reporting Items for Systematic Reviews and Meta-Analyses (PRISMA) reporting standards [28]. Operational terms and definitions were published a priori as part of the multicentre approach [3]; additional definitions are provided in Additional file 1: Appendix S2 and detailed in the PROSPERO registered protocol, including key questions, inclusion–exclusion criteria and a priori specified methods [29]. All protocol deviations and accompanying rationale are detailed in Additional file 1: Appendix S1.

### Search strategy and data sources

In consultation with an academic medical librarian (MVD), we iteratively developed a comprehensive search strategy using key papers and audit-improvement rounds to refine study catchment and feasibility [30]. The resulting health research partnership term clusters and the search strategy development methods have been applied to subsequent, parallel reviews [2, 3, 14, 15, 31]. We tested the strategy in Ovid MEDLINE to balance search sensitivity and scope [32]. The partnership search term cluster underwent peer review [33, 34] by an academic librarian to test for conceptual clarity across multiple partnership approaches. The overall strategy was subjected to the Peer Review of Electronic Search Strategies (PRESS) checklist review by a second academic network librarian, resulting in the spelling correction of a single term. No restrictions for date, design, language or data type were applied. The search strategy was translated for all four databases (Additional file 1: Appendix S3).

#### Electronic databases

Using the a priori, unrestricted strategy, we searched MEDLINE (Ovid), Embase, CINAHL Plus and PsycINFO from inception through 2 June 2021, including two updates. The search generated a total of 56 123 citations, resulting in the screening of 36 027 de-duplicated records [35] and 2784 full-text papers, managed with EndNote™ X7.8.

#### Eligibility and screening

We kept studies involving health research partnerships that (i) developed, used and/or assessed tools (or an element or property of a tool) to evaluate partnership outcomes or impacts [5, 36] as an aim of the study, and (ii) that also reported empirical evidence of tool psychometrics (e.g. validity, reliability). We excluded studies in which the main purpose of the partnership was recruitment and retention of study participants. Conference abstracts were excluded from the eligible literature only after full-text assessment or confirmation that the citations were preliminary or duplicate records, or were lacking sufficient abstraction detail [37]. Abstracts in languages other than English were passed through title/abstract (level 1 [L1]) screening but translated prior to full-text assessment (Table 1).

**Table 1:** Study inclusion–exclusion criteria

Inclusion criteria	Exclusion criteria
Include studies:	Exclude studies that:
(1) Pertaining to, describing or involving a health research partnership (inclusive of studies reporting evaluative, process evaluative, technical assistance and facilitated implementation research activity or roles)	(1) Do not meet the definition of a health research partnership
(2) Involving the development, use and/or assessment of a health research partnership outcome or impact assessment tool (or element/property of a tool), as an aim of the study (inclusive of multi-tool or toolkit studies, and studies involving frameworks/models when accompanied by a tool)	(2) Involve researcher–researcher or interprofessional (non-researcher inclusive) healthcare team partnerships
(3) Reporting empirical evidence of the psychometric properties of tools (e.g. validity, reliability)	(3) Do not involve the development, use and/or assessment of a health research partnership tool (or element/property of a tool), as an aim of the study
(4) That are accessible and amenable to full-text review	(4) Do not report empirical evidence of the psychometric properties of tools (e.g. validity, reliability)
(5) Reporting primary research findings drawn from empirical evidence	(5) Are not available or amenable to full-text review
(6) Reporting relevant abstractable data	(6) Report head-to-head tool comparisons without separately reporting tool-specific findings
(7) Of any design type that meets eligibility criteria	(7) Do not report primary research findings drawn from empirical evidence
	(8) Lack adequate or relevant abstractable data

All titles/abstracts (L1) and eligible full-text studies (L2) were screened and assessed independently, in duplicate (KJM with JB, LP, LN, SS, SM, MK, CM, AG, LS, KA), and tracked in a Microsoft (MS) Excel [38] citation database and screening spreadsheets. We tested and revised screening tools at each stage of the review and employed a minimum calibration rule (Cohen’s κ ≥ 0.60) [39] to align team members’ shared understanding of concepts and the application of eligibility criteria [40–43]. To balance abstraction burden with data availability and complexity, full-text abstraction (study and tool characteristics) was undertaken using a hybrid strategy [22, 44]. Eligible papers were independently abstracted by KJM and independently validated (MK, SS, SM, KP) [45] using a predefined coding manual. We resolved all discrepancies by consensus discussion [21, 41]. Investigators were sought out to locate missing tools or for assistance in differentiating linked citations only [43]. At least two attempts were made to locate corresponding authors and tools when contact details or tools were incorrect or missing [3, 5, 14]. The assessment and abstraction/scoring of psychometric, pragmatic tool evaluation and study quality characteristics were also undertaken independently and in duplicate, with discrepancies resolved the same way.

### Study and tool characteristics

Data pertaining to study and tool characteristics were abstracted per the protocol [29]. We anticipated challenges associated with consistent use of terminology as are commonly reported in this research domain (e.g. outcomes/impacts, partnership approaches, tool type) [3, 8, 14, 15]. When this occurred, we used the terms most prominent in methodological descriptions. We coded health subdomains inductively based on key words and study purposes [46]. More than one code per study was used to describe the study subdomain, as required.

### Empirical evidence of tool psychometrics

The empirical psychometric evidence for tools was evaluated for each identified tool. Informed by previous studies [6–12] and best-practice recommendations [17, 18, 36, 47, 48], we created an initial list of psychometric evidence types, and expanded this list iteratively when new sources were identified by included studies (Additional file 1: Appendix S3). Only studies reporting empirical psychometric evidence were retained in this review to (i) address the documented lack of research reporting psychometric evidence for health research partnership outcomes and impacts assessment tools, and (ii) advance our understanding about the presence and types of psychometric evidence available in existing literature beyond simple dichotomous labels (e.g. valid/not valid or reliable/not reliable). By synthesizing the presence of psychometric evidence across studies, we also aimed to highlight areas in which the nature and type of psychometric evidence could be improved and advance the science of partnership assessment. This approach necessarily focused on later testing and evaluation stages of tool development [49] but does not diminish the importance of conceptual and theoretical sources of evidence to establish tool reliability and validity as important precursor evidence sources. As previously reported, the identification and reporting of psychometric data was complex and varied substantially in level of detail. This was mitigated through iterative review, piloting and calibration; all abstraction discrepancies were independently, then collectively considered, then resolved to consensus through recurrent discussion.

### Pragmatic tool evaluation criteria

We modified a set of consensus-built criteria developed by Boivin et al. [7, 50] as an alternative to applying the Psychometric and Pragmatic Evidence Rating Scale (PAPERS) criteria [17, 18] due to the quality of reported data. The main purpose of the criteria checklist was to appraise the tools from the perspective of those intended to use the tools [7]. Team members iteratively modified and piloted the revised items. A final set of 20 criteria (five questions in four domains: Scientific Rigour, Partner Perspective, Comprehensiveness and Usability) were generated. Piloting confirmed that these criteria were a better fit for the level and detail present in the literature under examination, and provided a comprehensive, easily interpretable (single score) evaluation of scientific, partner, comprehensiveness and usability/accessibility properties for each tool (Additional file 1: Appendix S4). It is important to note that the original criteria were intended for use as a checklist, not a quality assessment [7]; we used them this way in our review. The modified criteria were applied independently and in duplicate to all tools [51], with discrepancies resolved by consensus. Tools were coded as toolkits in studies where multiple tools were described and intended for collective use; in these cases, tool characteristics were scored cumulatively and reported as a single tool.

### Study quality assessment: the quality of survey studies in psychology (Q-SSP) checklist [52]

Study quality assessments typically assess the degree to which adequate measures were taken to minimize bias and avoid errors throughout the research process [53], and are hence design-focused. After piloting several quality appraisal tools with the eligible literature, we found that the best-fitting tool was an assessment of survey methods, namely the Q-SSP appraisal checklist and guide (Additional file 1: Appendix S5). The Q-SSP checklist was developed to address a wide variety of research and to help investigators differentiate broadly acceptable from lower-quality studies [52] using a four-stage process comprising evidence review, expert consensus, checklist refinement and criterion validity testing [52]. Q-SSP assessments were undertaken independently, in duplicate, and we resolved discrepancies by consensus.

#### Analysis

Basic descriptive statics including means, standard deviations and frequencies were calculated to synthesize quantitative study, tool, psychometric and pragmatic characteristics in MS Excel [38] and Stata v13.1 software [54]. The synthesized data were consolidated into tables. Scores for each of the pragmatic and tool evaluation criteria (mean/standard deviation) were synthesized and reported by criterion, domain and overall sample. We synthesized qualitative variables using thematic analysis [46] in NVivo v12.7 [55], in keeping with the overarching descriptive-analytical approach for the review [56], and used existing reporting guidelines to organize the findings [57–59]. Finally, study quality assessments (Q-SSP) [52] were documented by calculating an overall quality (%) and four domain-specific scores (ratios) for each study.

## Results

**Fig. 1:**
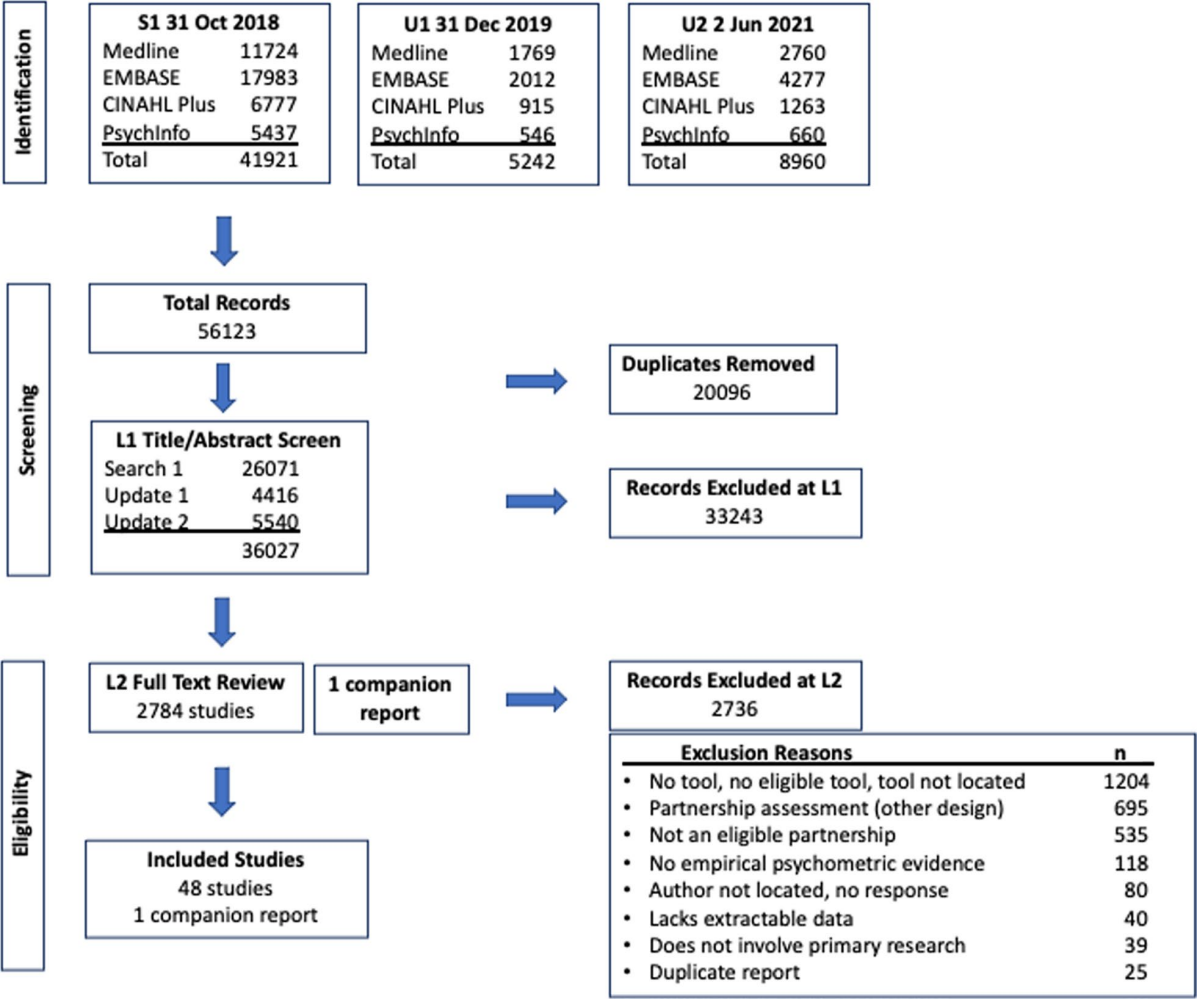
PRISMA systematic review study flow diagram

The search generated 36 071 de-duplicated records and 49 full-text studies (48 studies and one companion report), as depicted in the study flow diagram (Fig. 1).

The team Cohen’s kappa was 0.66 [95% CI (0.64–0.67)] at L1 title/abstract screening and 0.74 [95% CI (0.72–0.76)] at L2 full-text review; these results were categorized as “substantial” [39, 42].

### Study characteristics

Eligible studies comprised English-language and a single French-language report originating mostly in North America (39) and Europe (9), with a small remainder from South Africa (3), Australia (1) and Taiwan (1). Five dual-site studies involved the United Kingdom and South Africa (3), Canada and Australia (1), and Mexico and the United States (1) (Table 2).

**Table 2:** Characteristics of included studies (*n* = 48 studies, 1 companion report)

First author, year	Origin	Study design (methods)	Explicit conceptual foundation?	STUDY measures outcomes, impacts?	Contributions		
					Develop or modify tool	Use or evaluate tool	Validate^a^ tool
Butterfoss, 1996	North America (USA)	Mixed methods (MM)	B	O	✓	✓	✓
Kegler, 1998	North America (USA)	Cross-sectional study (Qn)	B	O	✓	✓	·
Chan, 2000	North America (USA)	Mixed methods (MM)	B	O	✓	✓	✓
Shortell, 2002	North America (USA)	Mixed methods (MM)	B	O	✓	✓	✓
Weiss, 2002	North America (USA)	Cross-sectional study (Qn)	B, G	O	✓	✓	✓
El Ansari, 2004	Europe (United Kingdom) Africa (South Africa)	Cross-sectional study (Qn)	B	O, I	✓	✓	✓
El Ansari, 2004	Europe (United Kingdom) Africa (South Africa)	Cross-sectional study (Qn)	B	O, I	✓	✓	·
Metzger, 2005	North America (USA)	Cross-sectional study (Qn)	B, G	O	✓	✓	✓
Kegler, 2005	North America (USA)	Cross-sectional study (Qn)	B	O, I	✓	✓	✓
Cramer, 2006	North America (USA)	Cross-sectional study (Qn)	B	O	✓	✓	✓
Feinberg, 2008	North America (USA)	Cross-sectional study (Qn)	B	O, I	·	✓	✓
Feinberg, 2008b	North America (USA)	Cross-sectional study (Qn)	B	O, I	·	·	✓
Orr Brawer, 2008	North America (USA)	Mixed methods (MM)	B, G	O, I	✓	✓	✓
King, 2009	North America (Canada)	Mixed methods (MM)	B	I	✓	✓	✓
Tolma, 2009	North America (USA)	Mixed methods (MM)	B	O	✓	✓	·
Wagemakers, 2010	Europe (Netherlands)	Multiple-case study (MM)	B	O	✓	✓	✓
King, 2010	North America (Canada)	Cross-sectional study (Qn)	B	O, I	·	✓	·
Ziff, 2010	North America (USA)	Cross-sectional study (Qn)	B	O	·	✓	✓
Jones, 2011	Europe (Ireland)	Mixed methods (MM)	B	O	✓	✓	✓
Jones, 2011b	Europe (Ireland)	Mixed methods (MM)	B	O	✓	✓	✓
Perkins, 2011	North America (USA)	Nested longitudinal study (MM)	B	O, I	·	✓	✓
Bilodeau (2008, 2019^b^	North America (Canada)	Mixed methods (MM)	B	O	✓	✓	✓
Curro, 2012	North America (USA)	Cross-sectional study (Qn)	·	O, I	✓	✓	✓
El Ansari, 2012	Europe (United Kingdom) Africa (South Africa)	Mixed methods (MM)	B	O	✓	·	✓
Brown, 2012	North America (USA)	Cross-sectional study (Qn)	B	O	✓	·	✓
Nargiso, 2013	North America (USA)	Cross-sectional study (Qn)	·	O	✓	✓	✓
Perkins, 2014	North America (USA)	Cross-sectional study (Qn)	B	O, I	✓	✓	✓
Chang, 2014	Australasia (Taiwan)	Post-test study (Qn)	B	O, I	✓	✓	✓
Brown, 2015	North America (USA)	Cross-sectional study (Qn)	B	O	·	✓	✓
Bornstein, 2015	North America (USA)	Cross-sectional study (Qn)	B	I	·	✓	·
Oetzel, 2015	North America (USA)	Nested cross-sectional study (Qn)	B	O, I	✓	✓	✓
Oetzel, 2015b	North America (USA)	Cross-sectional study (Qn)	B	O, I	·	·	✓
Stocks, 2015	Europe (United Kingdom)	Pre-post study (Qn)	B	O, I	✓	✓	✓
Brown, 2016	North America (Mexico, USA)	Cross-sectional study (Qn)	B	O	·	✓	✓
Goodman, 2017	North America (USA)	Cross-sectional study (Qn)	B	O	✓	✓	✓
Okazaki, 2017	North America (USA)	Case study (MM)	B	O, I	✓	✓	✓
Jones, 2018	Europe (Ireland)	Cross-sectional study (Qn)	B	O	✓	✓	✓
West, 2018	North America (USA)	Mixed methods (MM)	G	O	✓	✓	✓
Oetzel, 2018	North America (USA)	Multiple-case study (MM)	B	O, I	✓	✓	✓
Duran, 2019	North America (USA)	Mixed methods (MM)	B	O, I	·	✓	✓
Soobiah, 2019	North America (Canada)	Cross-sectional study (Qn)	B	O	✓	✓	✓
Dickson, 2020	North America (USA)	Cross-sectional study (Qn)	B	O	✓	✓	✓
Rodriguez Espinosa, 2020	North America (USA)	Mixed methods (MM)	B, G	O	✓	✓	✓
Lucero, 2020	North America (USA)	Cross-sectional study (Qn)	B	O	✓	✓	✓
van Schelven, 2021	Europe (Netherlands)	Mixed methods (Qn)	·	O, I	✓	✓	✓
Hamilton, 2021	North America (Canada)	Cross-sectional study (Qn)	B	O, I	✓	✓	✓
Boursaw, 2021	North America (USA)	Cross-sectional study (Qn)	B	O, I	✓	✓	✓
Loban, 2021	North America (Canada) Australasia (Australia)	Cross-sectional study (Qn)	B	O, I	✓	✓	✓

(B) informed by conceptual theory, model, framework; (G) generated conceptual theory, model, framework; (·) conceptual underpinnings not explicitly identified

*Qu* qualitative study, *Qn* quantitative study, *MM* mixed-methods study, *O* outcomes, *I* impacts

^a^Empirical validation

^b^Companion report

**Fig. 2:**
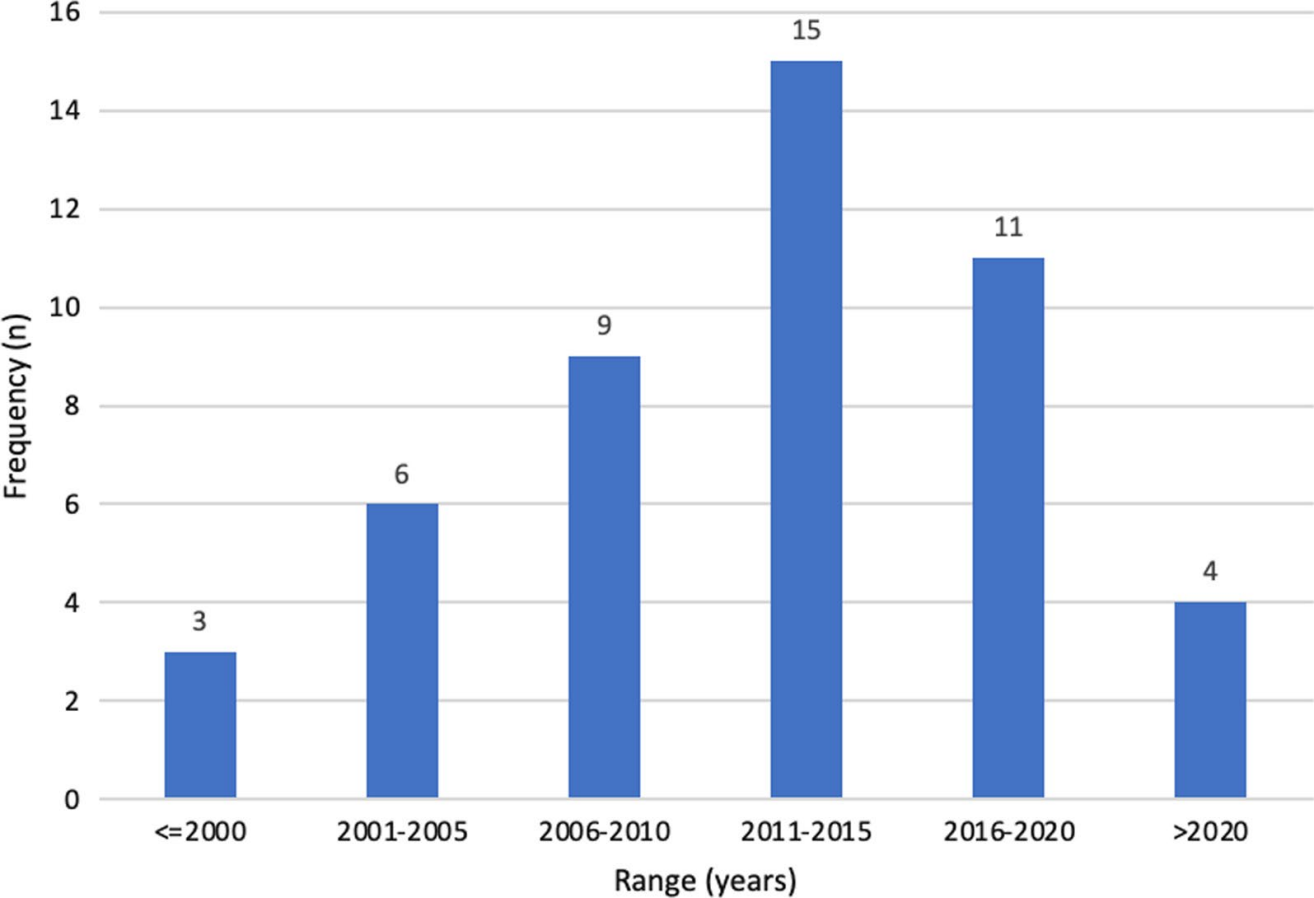
Year of publication for included studies (*n* = 48 studies)

The eligible literature was widely dispersed, with exactly half of the publications (24, 50%) published in the same number of journals. Several small publication clusters were identified, including seven studies in *Health Education & Behaviour* (15%), three each in the *American Journal of Community Psychology*, *Global Health Promotion* and theses (each 6%), and two each in *Health Promotion International*, *Public Health Nursing*, *Evaluation and Program Planning* and *Health Promotion Practice* (each 4%). As shown in Fig. 2, about half of the identified literature was published after 2014 (20, 42%), and the earliest study was published in 1996.

Most studies involved cross-sectional (28, 58%) and mixed methods with embedded survey (14, 29%) designs, case/multi-case (3, 6%), post- and pre-post designs (2, 4%), and a single nested longitudinal study (1, 2%) (Table 2). Studies employed quantitative (31, 65%) or mixed methods (17, 35%), and of the mixed-methods studies (17), most were true mixed quantitative–qualitative methods (14, 82%), and the remainder were mixed qualitative (2, 12%) and mixed quantitative (1, 6%) methods.

**Fig. 3:**
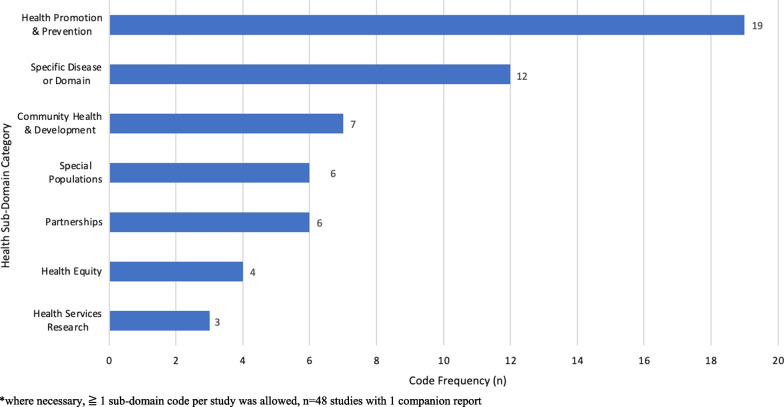
Health subdomain clusters

The studies were conducted in multiple health subdomains (Fig. 3), including health promotion, prevention and public health (19), and disease-specific domains [i.e. cancer, mental health and substance use/harm reduction, and sexually transmitted/blood-borne infections and sexual health (12)]. The smaller subdomains included community health and development (7), special populations (e.g. primary care, paediatric/adolescent health, and immigrant and geriatric health) (6), partnerships (6), health equity (4) and health services research (3).

Most studies reported explicit conceptual underpinnings (44, 91%). Methodologically, studies were multifocal, contributing to the health research partnership assessment literature through tool validation (44, 92%), development (25, 52%), modification (21, 44%) and evaluation (13, 27%), and measured outcomes (25, 52%), impacts (2, 4%) or both outcomes and impacts simultaneously (21, 44%). Explicit definitions for the terms outcome and impact were available in less than half of studies (20, 42%), and terms were frequently switched.

### Tool characteristics

Included studies yielded 58 tools. The characteristics of the included tools are summarized in Table 3. With one exception, studies were exclusively English-language, and six contained non-English-language tools (English–Spanish, 3 [60–62]; English–French, 2 [63–65]; and Dutch, 1) [66]). Tools targeted multiple partner groups including partnership members (28, 43%), community members (11, 17%), researchers (10, 15%), patients, and public and coalition staff (4, 6% respectively), and to a lesser extent targeted research staff (3, 5%), healthcare staff and partner organizations (2, 3% respectively), and education staff members (1, 2%). Surveys (21, 36%), questionnaires (17, 29%) and scales (12, 21%) were the most common tool types identified, and these categories were complicated by frequent switching of terms (survey, questionnaire, scale) and variable categorization across reports. We also identified several toolkits (3, 5%), indices and rubrics (2, 3%, respectively), and a single checklist (2%).

**Table 3:** Tool characteristics (*n* = 58 tools)

Tool name	Tool purpose	Targeted partners	TOOL measures outcome (O), process (P), impact (I)?	Empirical psychometric evidence for: validity (V), reliability (R)		Tool evaluation score (%)	First author, year
				V	R		
The Committee Member Survey (CMS)^a^ The Plan Quality Index (PQI)^a^	CMS: to measure committees’ work and effectiveness PQI: to measure committee plan quality	Organizational committee members	O, P	✓	✓	60	Butterfoss, 1996
questionnaire	To address coalition factors specified in the conceptual model and two outcomes: member satisfaction and participation	Coalition members	O, P	✓	✓	65	Kegler, 1998
Social Capital Index (scale adapted from the Partnership Self-Assessment Survey, Health Research & Educational Trust [103])	To assess institutional social capital as a functional relationship based on trust, involvement and reciprocity among partners in a network	Partnership members	O, P	✓	·	35	Chan, 2000
Capability Index [Partnership Self-Assessment Survey (PSAS)]	To assess the perceived effectiveness of the partnership	Coalitions	O, P	✓	✓	45	Shortell, 2002
Partnership Self-Assessment Tool	To measure partnership synergy and functioning dimensions, and gather descriptive info about partnerships and partners	Partnership coordinators Partners	O, P	✓	✓	80	Weiss, 2002
survey	To examine partnership involvement in operational aspects and compare perceived benefits, costs, satisfaction, commitment and ownership	Partnership members	O, P, I	✓	✓	65	El Ansari, 2004
Partnership Member Survey	To assess nurses’ perceptions of 17 aspects of partnership functioning in the formation and implementation of community partnerships	Health services staff (nurses)	O, P, I	·	✓	50	El Ansari, 2004b
Partnership Self-Assessment Survey (PSAS)-derived scales	To assess coalition participants' perceptions about coalition decision-making, conflict management, leadership and culture, and the effectiveness of their coalition in goal attainment, participation level and perceived participation costs/benefits	Coalition members	O, P	✓	✓	45	Metzger, 2005
Coalition Member Survey	To assess participants’ perceptions about the coalition and the project; to describe the relationship between partnership dimensions and both interim and community-wide outcomes, as perceived by coalition members	Coalition members [members from 13 communities funded by the Centers for Disease Control and Prevention (CDC)]	O, P, I	✓	✓	65	Kegler, 2005
Internal Coalition Effectiveness (ICE) Instrument	To measure outcomes and identify organizational strengths and areas for improvement	Coalition partners (leaders, members of public and private agencies)	O, P	✓	✓	55	Cramer, 2006
CTC Coalition Web-Based Self-Report Questionnaire	To determine whether coalition functioning or community characteristics predict either sustained CTC activity or new funding	Coalition members Coalition staff Coalition facilitators	O, P, I	✓	✓	55	Feinberg, 2008
CTC Coalition Web-Based Self-Report Questionnaire	To assess coalition functioning as a research tool and as part of a feedback system for technical assistance providers and coalition members	Coalition participants Technical facilitation staff	O, P, I	✓	✓	65	Feinberg, 2008b
Partnership Self-Assessment Tool (PSAT) [80] Social Capital Survey [104, 105, 106]	PSAT: to assess synergy and partnership function Social Capital Survey: to assess trust between members and organizations, elements of reciprocity and perceptions of the value of membership, and social capital (individual, aggregated community) levels, and individual, organizational and community empowerment and control	Coalition members	O, P, I	✓	✓	75	Orr Brawer, 2008
				✓	✓	60	
Community Impacts of Research Oriented Partnerships (CIROP)	To measure community members’ perceptions of the impact of research partnerships addressing health or social issues	Partnership members [healthcare professionals (HCP), managers, researchers, teachers, principals]	I	✓	✓	75	King, 2009
Profile of Collaboration Survey [107]^a^	To assess stakeholder satisfaction with facets and results of collaboration	Stakeholders, evaluators	O, P	·	✓	60	Tolma, 2009
Coordinated Action Checklist	To evaluate and facilitate coordinated action in community health promotion partnerships	Partnership members	O, P	✓	✓	75	Wagemakers, 2010
• Community Impacts of Research Oriented Partnerships (CIROP) Questionnaire • Background Information Form for Research Partnerships • Research Contact Checklist • CIROP Respondent Form	CIROP questionnaire: To measure community members’ perceptions of medium-term impacts of partnerships addressing health or social issues Background Information Form for Research Partnerships: to assess partnership purpose, structural and functional features, outputs, people and organizations Research Contact Checklist: To capture community members’ requests CIROP Respondent Form: to capture respondents’ awareness of the partnership’s purpose, products and information-sharing, their relationship with the partnership and characteristics	Community members	O, P, I	✓	✓	80	King, 2010
Wilder Collaboration Factors Inventory [108]	To assess the presence of successful collaboration factors in a partnership, organized by six partnership assessment domains	Coalition staff community members	O, P	✓	✓	55	Ziff, 2010
Jones Synergy Scale	To measure synergy in health promotion partnerships	Partnership members	O, P	✓	✓	50	Jones, 2011
Jones Trust Scale	To measure partnership trust and partnership functioning in health promotion partnerships	Health promotion partners	O, P	✓	✓	45	Jones, 2011b
CTC Websurvey for Agency Directors, Team Members Web-Based Survey for Technical Assistants	CTC Websurvey: to assess perceptions about team functioning, individual, workplace and community characteristics Web-Based Survey: to assess team dynamics perceived by technical assistants	Community members Organizational administrators Technical staff	O, P, I	✓	✓	45	Perkins, 2011
				✓	✓	50	
Self-Evaluation Tool for Action in Partnership [109, 110, 110] French and English Versions	To assess partnership functioning by identifying difficulties and aspects that work well using six effectiveness requirements	Partnership members	O, P	·	✓	85	Bilodeau, 2011, 2019^b^
Survey	To assess the benefits and challenges of participation in the PEARL [Practitioners Engaged in Applied Research & Learning] practice-based research network	Clinician researchers	O, I	·	✓	50	Curro, 2012
Survey	To explore partnership members' perceived internal, external, organizational, personnel features and outcomes	Partnership members (partnership staff, health and social services staff and clinicians, academics)	O, P	✓	✓	65	El Ansari, 2012
CTC Coalition Web-Based Survey	To assess internal coalition functioning, including leadership, interpersonal relationships, task focus, participation benefits/costs, and sustainability planning and external coalition functioning	Coalition members Technical assistance providers	O, P	✓	✓	65	Brown, 2012
General Coalition Capacities Scale General Coalition Capacity Rubric Environmental Strategy (ES)-Specific Capacity Rubric	CLI: to quantitatively measure general coalition functioning GCS: to measure coalition leadership, membership/staff turnover, meeting quality, level of community visibility, technological capacity ES-specific capacity scale: to measure ES implementation capacity	Coalition members [grant coordinator or designee from each site; experts, state-level prevention professionals (HCP)]	O, P	✓	·	45	Nargiso, 2013
				·	✓	40	
				·	✓	45	
Adapted survey [based on PSAT(S) [112, 113; 114]	To assess individual demographic, institutional, partnership and sustainability factors of academic practice partnerships	Advanced practice partners (affiliated with nursing, corporations, government, foundations, researchers, meeting attendees, nursing leaders, academic practice partnership participants)	O, P, I	✓	✓	60	Perkins, 2014
Taiwan Health Promotion in Schools (HPS) Support Network Evaluation Study Survey	To measure health promotion in schools (HPS) implementation and impact, and efficacy of implementation	Teachers, director, section chief, school nurse	O, P, I	✓	·	60	Chang, 2014
• CTC Member Coalition Function Survey • CTC Functioning Survey [Pennsylvania Commission on Crime and Delinquency (PCCD) technical assistance providers] • Coalition function survey supplement K • Coalition function survey supplement L	To assess coalition function in three domains (collaborative processes, coalition capacities, coalition activities)	Coalition partners Coalition technical assistance providers Mobilizers, voluntary chairs	O, P	✓	✓	70	Brown, 2015
				✓	✓	65	
				✓	✓	65	
				✓	✓	65	
Member Involvement in Physical Activity Coalitions (MIPAC) Survey	To measure organizational representatives’ perspectives about the characteristics of physical activity (PA) coalitions, the characteristics of organizational members, factors related to organizational member involvement, and perceived PA coalition success	Coalition members (nonprofit, for profit and government agency representatives)	I	✓	✓	70	Bornstein, 2015
Key Informant Survey (KIS) Community Engagement Survey (CES)	KIS: To gather relevant information about projects and identify academic/community partners CES: to assess perceptions of context, processes and outcomes using the CBPR conceptual model	Researchers Researchers academic Partners Community partners	O, P, I	✓	✓	85	Oetzel, 2015
				✓	✓	85	
Validation of 22 scales in Community Engagement Survey (CES)	To assess perceptions of context, processes and outcomes using a CBPR conceptual model	Academics HCP Community members	O, P, I	✓	✓	85	Oetzel, 2015b
questionnaire (adapted from Morrow et al. [115])	To quantitatively evaluate quality of the PPI within the research user group (RUG) that may be generalized to other settings	Research user group	O, P, I	✓	✓	85	Stocks, 2015
Coalition Context and Capacity Assessment Survey	To assess coalition context and capacity constructs and gain a better understanding of how to improve substance abuse prevention by community coalitions	Coalition partners	O, P	✓	✓	70	Brown, 2016
Community Engagement Measure	To assess how engagement principles were adhered to by partnership members	Community member co-research trainees	O, P	·	✓	90	Goodman, 2017
Coordinating Council Member Survey	To document members’ perception of the effectiveness of—and satisfaction with—adapting and implementing evidence-based health programmes based on the principles of effective partnerships	Coalition staff	O, P, I	·	✓	45	Okazaki, 2017
Partnership survey	Partnership survey: To assess the contribution of factors that influence partnership trust and mistrust	Partnership members	O, P	✓	✓	65	Jones, 2018
Scale of Perceived Trustworthiness	To assess each respondent’s perception of their research partners’ trustworthiness	Research partnership members (community-based organization members, leaders, advisory boards, individuals in partnership roles; community partners, academic partners with dual roles, conference attendees, researchers)	O, P	✓	✓	60	West, 2018
Selected scales from: • Key Informant Survey (KIS) • Community Engagement Survey (CES)	KIS: to gather relevant information about projects and identify academic and community partners CES: to explore perceptions of context, processes and outcomes corresponding to a community-based participatory research conceptual model	Researchers Researchers, community members	O, P, I	✓	✓	90	Oetzel, 2018
				✓	✓	90	
• Key Informant Survey (KIS) • Community Engagement Survey (CES)	To assess research context, process and outcome measures, including measures of power/resource-sharing and structural characteristics of projects	Researchers Academics community partners	O, P, I	✓	✓	90	Duran, 2019
				✓	✓	90	
Modified Patient Engagement Evaluation Tool (PEET) [116]	To quantify engagement using evidence-informed criteria from the stakeholder engagement in comparative effectiveness research framework	Patients Caregivers Geriatricians	O, P	✓	✓	75	Soobiah, 2019
Key Informant Survey (KIS) (English and Spanish translation versions)	To capture key “factual” information about the project and partnership that could be identified by a principal investigator or designate	Researchers Community members	O, P	✓	✓	90	Dickson, 2020
CBPR Processes and Practices, and outcomes scales (from E2 Key Informant (KIS) and Community-engagement Surveys (CES)	KIS: to gather collected project-related information (e.g. funding dates, financial resource-sharing and use of formal agreements) CES: to obtain perceptions of CBPR model constructs including partnership processes (relational and structural dynamics) and individual and project outcomes	Community partners Academic partners	O, P	✓	✓	90	Rodriguez Espinosa, 2020
CBPR Process Scales (synergy, trust, CBPR principles, participation, influence) and Trust Typology [from E2 Community Engagement Survey (CES)]	To test the quantitative structural elements of the trust typology, identify variability in trust correlates, and create an empirical foundation for the trust types	Researchers Academic partners Community partners	O, P	✓	✓	85	Lucero, 2020
Project Outcome Scale	To assess the self-perceived contribution of projects to the social position of young persons with chronic conditions	Patients	O, P, I	✓	✓	70	Van Schelven, 2021
Patient Engagement in Research Scale (PEIRS-22 shortened version) (modified from Hamilton et al. [117])	To assess the degree of meaningful engagement of patients and family caregivers as partners in research projects	Patients, family caregivers	O, P, I	✓	✓	75	Hamilton, 2021
Community Engagement Survey [scales (7) with subscales (23)]	To assess researcher and community member perceptions of CBPR contexts, mechanisms and outcomes	Researchers, community members	O, P, I	✓	✓	85	Boursaw, 2021
IMPACT [Innovative Models Promoting Access-to-care Transformation] Partnership Questionnaire [80, 86]	To assess partnership functioning and synergy	Partnership stakeholders	O, P, I	✓	✓	60	Loban, 2021

(·) conceptual underpinnings not explicitly identified

*CBPR* community-based participatory research, *CTC* Communities That Care, *PPI* public and patient involvement

^a^Psychometric data noted for multiple toolkit tools

^b^Companion report

Almost all tools assessed process (55, 95%), but only half assessed outcomes (30, 52%) or both outcomes and impacts (26, 45%). Very few focused on impact assessment alone (2, 3%); however, we observed inconsistencies in the use and definition of these terms. We identified multiple forms of empirical evidence for validity (86%, 50) and reliability (95%, 55) in the tools. The presence of conceptual underpinnings (90%, 52) was the same as study-level conceptualization.

### Pragmatic tool evaluation scores

Tables 4 and 5 present a synthesis of pragmatic tool evaluation criteria [7] (Additional file 1: Appendix S4). Mean domain scores were highest for *Comprehensiveness* (3.79, SD 0.75) and *Scientific Rigour* (3.58, SD 0.87), followed by *Usability* (3.19, SD 1.38). The lowest mean domain score was for *Partner Perspective* (2.84, SD 1.04), which was a surprising finding given the review focus on health research partnership assessment.

**Table 4:** Pragmatic tool evaluation consolidated scores (*n* = 58 tools)

Tool evaluation criteria		Present (*n*)	Absent (*n*)	Cannot answer (*n*)	Score (%)
Scientific rigour					
SR1	Based on systematic literature review?	10	41	7	17%
SR2	Based on the experiences or expertise of partners?	32	25	1	55%
SR3	Based on a conceptual or theoretical framework?	52	6	·	90%
SR4	Is there evidence of tool validity? (any source)	52	6	·	90%
SR5	Is there evidence of tool reliability? (any source)	54	4	·	93%
Scientific rigour domain score			Mean 3.58	Std dev 0.87	Range 1–5
Partner perspective					
PP1	Partners involved as co-designers?	34	23	1	59%
PP2	Tool designed to be self-administered by partners?	56	2	·	97%
PP3	Assessment results must be reported back to partners?	16	41	1	28%
PP4	Tool assesses level of partner involvement?	16	42	·	28%
PP5	Tool captures the influence of partners?	44	14	·	76%
Partner perspective domain score			Mean 2.84	Std dev 1.04	Range 1–5
Comprehensiveness					
C1	Tool documents partnership context?	56	2	·	97%
C2	Tool assesses partnership process?	55	3	·	95%
C3	Tool documents partnership outcome(s) and/or impact(s)?	58	0	·	100%
C4	Tool monitors the partnering process at multiple moments?	19	37	2	33%
C5	Tool consists of open- and closed-ended questions?	32	25	1	55%
Comprehensiveness domain score			Mean 3.79	Std dev 0.75	Range 2–5
Usability					
U1	Tool purpose stated?	58	0	·	100%
U2	Tool freely accessible?	29	29	·	50%
U3	Tool available in a readily usable format?	36	22	·	62%
U4	Tool easy to read and understand?	31	26	1	53%
U5	Tool accompanied by instructions?	33	25	·	57%
Usability domain score			Mean 3.19	Std dev 1.38	Range 1–5
Overall D1–D4 total score			Mean 66.64	Std dev 15.54	Range 35–90

(·) conceptual underpinnings not explicitly identified

**Table 5:** Health research partnership tool evaluation—study scores (*n* = 48 with 1 companion report; *n* = 58 tools)

First author, year	Tool name	Reported tool type	Domain scores^a^				Tool score (%)
			SR	PP	C	U	
Butterfoss, 1996	• Committee Member Survey (CMS) • The Plan Quality Index (PQI)	Toolkit	3	3	3	3	60
Kegler, 1998	• questionnaire	Questionnaire	3	3	4	3	65
Chan, 2000	• Social Capital Index (scale adapted from the Partnership Self-Assessment Survey, Health Research & Education Trust, 1997)	Index	2	1	3	1	35
Shortell, 2002	• Capability Index [from Partnership Self-Assessment Survey (PSAS), Health Research & Educational Trust 1997]	Index	3	2	3	1	45
Weiss, 2002	• Partnership Self-Assessment Tool (PSAT)	Questionnaire	5	4	4	3	80
El Ansari, 2004	• survey	Survey	3	4	3	3	65
El Ansari, 2004	• Partnership Member Survey	Survey	2	3	3	2	50
Metzger, 2005	• Partnership self-assessment survey (PSAS)-derived scales	Scales	3	2	3	1	45
Kegler, 2005	• Coalition Member Survey	Survey	3	4	4	2	65
Cramer, 2006	• Internal Coalition Effectiveness (ICE) Instrument	Survey	4	2	3	2	55
Feinberg, 2008	• CTC Coalition Web-Based Self-Report Questionnaire	Questionnaire	3	2	5	1	55
Feinberg, 2008b	• CTC Coalition Web-Based Self-Report Questionnaire	Questionnaire	3	4	5	1	65
Orr Brawer, 2008	• Partnership Self-Assessment Tool (PSAT) [80]	Questionnaire	2	4	4	5	75
	• Social Capital Survey [104, 105, 106]	Survey	3	2	3	4	60
King, 2009	• Community Impacts of Research-Oriented Partnerships (CIROP)	Questionnaire	4	2	4	5	75
Tolma, 2009	• Profile of Collaboration Survey [107] • Interactive group evaluation form • Meeting observation form • Facilitator check-off list • Random Electronic Survey	Toolkit	2	3	4	3	60
Wagemakers, 2010	• Coordinated Action Checklist	Checklist	5	2	4	4	75
King, 2010	• Community Impacts of Research-Oriented Partnerships (CIROP) Questionnaire • Background Information Form for Research Partnerships • Research Contact Checklist • CIROP Respondent Form	Toolkit	4	2	5	5	80
Ziff, 2010	• Wilder Collaboration Factors Inventory [108]	Questionnaire	3	1	4	3	55
Jones, 2011	• Jones Synergy Scale	Scale	4	2	2	2	50
Jones, 2011b	• Jones Trust Scale	Scale	3	2	2	2	45
Perkins, 2011	• CTC Web-survey for Agency Directors, Team Members	Questionnaire	3	1	4	1	45
	• Web-Based Survey for Technical Assistants	Questionnaire	3	2	4	1	50
Bilodeau (2011, 2019^b^)	• Self-Evaluation Tool for Action in Partnership/L'Outil diagnostique de l'action en partenariat [Bilodeau et al. 109, 110, 111, 64) French and English Versions	Questionnaire	4	4	4	5	85
Curro, 2012	• survey	Survey	2	3	3	2	50
El Ansari, 2012	• survey	Survey	4	3	3	3	65
Brown, 2012	• CTC Coalition Web-Based Survey	Questionnaire	3	3	5	2	65
Nargiso, 2013	• General Coalition Capacities Scale	Scale	2	1	4	2	45
	• General Coalition Capacity Rubric	Rubric	2	1	3	2	40
	• Environmental strategy-specific capacity rubric	Rubric	2	2	3	2	45
Perkins, 2014	• Adapted survey [based on PSAT(S) [112, 113; 114]	Survey	3	2	4	3	60
Chang, 2014	• Taiwan Health Promotion in Schools (HPS) Support Network Evaluation Study Survey	Survey	3	3	3	3	60
Brown, 2015	• CTC Member Coalition Function Survey	Questionnaire	3	3	5	3	70
	• CTC Functioning Survey [Pennsylvania Commission on Crime and Delinquency (PCCD) technical assistance providers]	Questionnaire	3	3	4	3	65
	• Coalition function survey supplement K (mobilizers, voluntary chairs only)	Questionnaire	3	2	5	3	65
	• Coalition function supplement L (mobilizers, voluntary chairs only)	Questionnaire	3	2	5	3	65
Bornstein, 2015	• Member Involvement in Physical Activity Coalitions (MIPAC) Survey	Survey	4	3	3	4	70
Oetzel, 2015	• Key Informant Survey (KIS)	Survey	4	4	4	5	85
	• Community Engagement Survey (CES)	Survey	4	4	4	5	85
Oetzel, 2015b	• Community Engagement Survey (CES)	Survey	4	4	4	5	85
Stocks, 2015	• questionnaire (adapted from Morrow et al. [115])	Questionnaire	4	4	5	4	85
Brown, 2016	• Coalition Context and Capacity Assessment Scales (from CTC Coalition Web-Based Survey)	Scales	3	3	4	4	70
Goodman, 2017	• Community Engagement Measure	Survey	5	5	4	4	90
Okazaki, 2017	• Coordinating Council Member Survey	Survey	2	3	3	1	45
Jones, 2018	• Trust, Mistrust and Power scales from the Partnership Survey	Scales	4	2	4	3	65
West, 2018	• Scale of Perceived Trustworthiness	Scale	4	2	3	3	60
Oetzel, 2018	Scales from: • Key Informant Survey (KIS)	Survey	5	4	4	5	90
	Scales from: • Community Engagement Survey (CES)	Survey	5	4	4	5	90
Duran, 2019	• Constructs and sub-constructs (context and partnership processes domains) and outcomes domains from the Community Engagement Survey (CES)	Survey	5	4	4	5	90
	• Constructs and sub-constructs (context and partnership processes domains) and outcomes domains from the Key Informant Survey (KIS)	Survey	5	4	4	5	90
Soobiah, 2019	• Modified Patient Engagement Evaluation Tool (PEET) [116]	Survey	4	3	4	4	75
Dickson, 2020	• Items from the Key Informant Survey (E2 KIS) (English and Spanish translation versions)	Survey	4	5	4	5	90
Rodriguez Espinosa, 2020	• CBPR processes & practices, and outcomes scales [from E2 Key Informant (KIS) and Community-engagement Surveys (CES)]	Scales	5	4	4	5	90
Lucero, 2020	• CBPR Process Scales (synergy, trust, CBPR principles, participation, influence) and Trust Typology [from E2 Community Engagement Survey (CES)]	Scales/typology	4	3	5	5	85
van Schelven, 2021	• Project Outcome Scale	Scale	3	4	4	3	70
Hamilton, 2021	• Patient Engagement in Research Scale (PEIRS-22 shortened version) (modified from Hamilton et al. [117])	Scale	4	3	3	5	75
Boursaw, 2021	• Community Engagement Survey (CES) scales	Scales	5	3	4	5	85
Loban, 2021	• IMPACT [Innovative Models Promoting Access-to-care Transformation] Partnership Questionnaire [80, 86﻿]	Questionnaire	3	2	4	3	60

*SR* Scientific Rigour domain, *PP* Partnership Perspective domain, *C* Comprehensiveness domain, *U* Usability domain

^a^Domain scores from the Health Research Partnership Tool Evaluation Criteria (adapted from Boivin et al. [7]) (see Additional file 1: Appendix S4)

^b^Companion paper

Tool  *comprehensiveness* was high in terms of documenting outcomes and/or impacts (100%), partnership process (95%) and context (97%); however, tools lacked deliberate design for recurrent monitoring of partnerships (33%).

In terms of *Scientific Rigour*, tools were not typically informed by systematic evidence (17%) but were conceptually grounded (90%) and presented evidence for both validity and reliability (90% and 93%, respectively, inclusive of both empirical and theoretical/conceptual sources). Only half of the tools were explicitly based on the experiences and expertise of partners (55%).

Overall, tool *Usability* was mixed. Tool purpose was always present (100%), but only half of the tools were freely accessible (50%), considered easy to read and understand (53%), accompanied by instructions (57%) and available in a readily usable format (62%).

Tools were generally designed to be self-administered (97%), but not for reporting back to partners (28%). The level of partner involvement was not commonly included (28%), and partners were deliberately involved as co-designers in only 59% of studies, despite frequent capture of partner influence (76%).

**Fig. 4:**
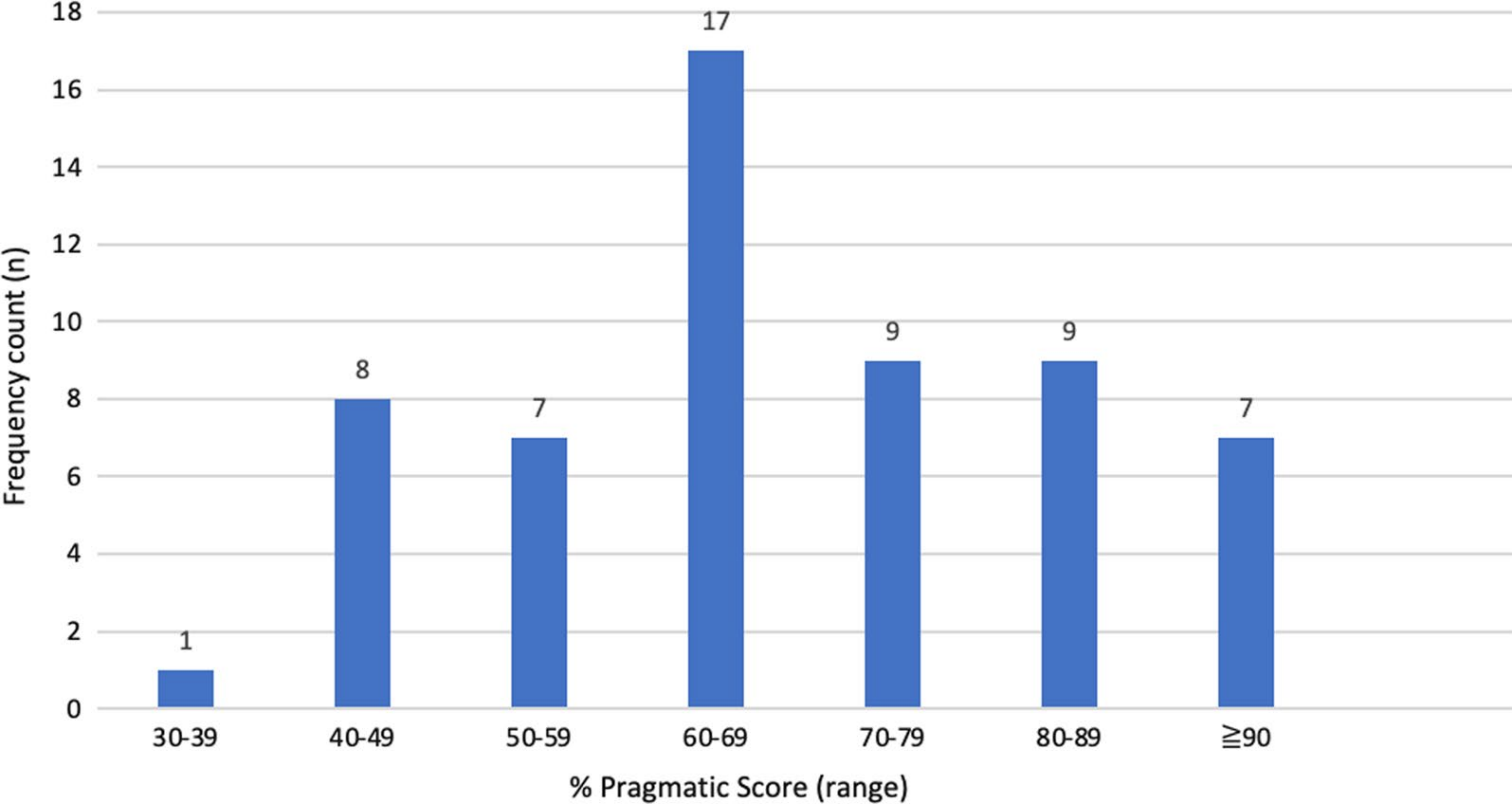
Pragmatic tool assessment—criteria total scores (*n* = 58 tool scores)

The overall tool evaluation mean score was 66.64 (SD15.54), with scores ranging from 35 to 90% (Fig. 4).

The domains and total score analysis highlighted strengths for several tools. Twelve tools scored high (4 or 5) across all four domains (≥ 85%) [61–64, 67–72], and an additional two tools [73, 74] had lower *Partner Perspective* domain scores (3) but still achieved a high total score (85%) across the remaining three domains. Several tools demonstrated top scores for *Comprehensiveness* [69, 73, 75–79] while others scored higher in *Scientific Rigour* [61, 66, 70–72, 74, 80] and *Usability* [61–64, 67, 68, 71–74, 77, 81–83]. Few achieved top scores in the *Partner Perspectives* domain [62, 70] (Tables 4 and 5).

### Psychometric assessment

Psychometric testing and reporting were widely variable and challenging to assess, primarily due to inconsistent or incomplete testing, reporting and reporting detail. Almost three quarters of studies presented two or more forms of psychometric evidence for validity (35, 73%); eight studies (17%) presented two forms of evidence for reliability. Iterative assessment and abstraction of psychometric evidence revealed reliability evidence in four categories (internal consistency, test–retest reliability, inter-rater reliability and other). The most frequently occurring form of reliability evidence was internal consistency (83%). Validity evidence was found in 11 categories [construct validity (convergent, factorial, discriminant, known groups, other), criterion validity (predictive, concurrent), structural validity (dimensionality), responsiveness, face validity, and content validity] (Table 6). The most frequent validity evidence was convergent construct validity (43, 27%) and predictive criterion validity (31, 20%). We observed norms and abstracted two forms of evidence for interpretability (ceiling/floor effects and interpretability); however, both evidence forms were rare.

**Table 6:** Consolidated tool psychometric evidence (*n* = 58 tools)

Psychometric criteria	Code frequency (*n*)	Frequency (%)
**Reported evidence of reliability**	**64**	**100**
Internal consistency	53	83
Test–retest reliability	5	8
Inter-rater reliability	4	6
Reliability (other)	2	3
**Reported evidence of validity**	**158**	**100**
Construct validity (convergent)	43	27
Criterion validity (predictive)	31	20
Criterion validity (concurrent)	14	9
Construct validity (factorial)	14	9
Content validity	14	9
Structural validity (dimensionality)	12	8
Face validity	10	6
Construct validity (known groups)	5	3
Construct validity (other)	3	2
Responsiveness	3	2
**Reported evidence of norms**	**2**	**100**
**Reported evidence of interpretability**	**4**	**100**
Ceiling and/or floor effects	3	75
Interpretability (other)	1	25

Each of the bolded lines denotes the overarching category of psychometric evidence described. Lines beneath each bolded category present the respective types of reliability, validity, norms and interpretability we identified from the selected literature

We identified 18 studies with more advanced and comprehensive assessment and reporting of psychometric evidence for validity and reliability [60, 61, 65, 68, 69, 71, 72, 74, 78–80, 82–88]; several of these studies overlapped with high-scoring tools identified using pragmatic tool evaluation criteria [61, 68, 69, 71, 72, 74].

### Study quality assessment (Q-SSP)

The Q-SSP assessment revealed an overall mean study quality score of 58.02% (SD 12.32%), with scores ranging from 25 to 80%. Most studies (42, 88%) scored < 75%, and thus were categorized as having “questionable” quality by convention; very few studies (6, 12%) scored ≥ 75% or within the “acceptable” range [61, 65, 71, 81, 88, 89] (Table 7).

**Table 7:** Q-SSP assessments by item, domain and total score for included studies (*n* = 48 studies, 1 companion report)

First author, year	Q1	Q2	Q3	Q4	Q5	Q6	Q7	Q8	Q9	Q10	Q11	Q12	Q13
Butterfoss, 1996	1	1	1	0	0	1	0	0	1	1	0	0	0
Kegler, 1998	1	NC	0	1	1	0	0	0	0	1	0	0	0
Chan, 2000	1	1	1	1	1	1	NC	0	0	1	1	1	0
Shortell, 2002	1	1	1	1	0	0	NC	0	0	1	1	1	0
Weiss, 2002	1	1	1	1	1	1	0	0	0	1	1	1	0
El Ansari, 2004	1	1	1	1	0	0	0	0	0	1	1	1	0
El Ansari, 2004b	1	1	0	0	1	1	0	0	0	1	0	1	0
Metzger, 2005	1	NC	1	0	1	1	0	1	1	1	0	1	0
Kegler, 2005	1	1	0	1	1	1	0	0	1	1	0	0	0
Cramer, 2006	1	NC	0	1	0	1	0	0	0	1	1	1	0
Feinberg, 2008	1	1	1	0	1	1	0	0	1	1	0	1	0
Feinberg, 2008b	1	1	1	1	0	1	0	0	0	1	1	1	1
Orr Brawer, 2008	1	1	1	1	1	1	NC	NC	1	1	1	0	0
King, 2009	1	1	1	0	1	1	0	0	0	1	1	1	0
Tolma, 2009	1	NC	0	0	0	0	0	0	0	NC	0	0	1
Wagemakers, 2010	1	1	0	0	1	1	1	0	0	1	1	1	0
King, 2010	1	1	0	1	1	1	0	0	0	1	1	1	0
Ziff, 2010	1	1	0	1	1	1	0	0	0	1	0	1	0
Jones, 2011	1	1	1	1	0	1	0	0	0	1	1	1	0
Jones, 2011b	1	1	0	1	1	1	NC	0	NC	1	1	1	0
Perkins, 2011	1	1	1	1	1	1	0	0	0	1	0	1	1
Bilodeau, 2011 and 2019^b^	1	1	0	1	1	0	0	0	0	NC	1	1	1
Curro, 2012	1	1	0	0	1	1	0	0	0	1	0	0	0
El Ansari, 2012	1	1	0	1	1	1	0	0	0	1	1	0	0
Brown, 2012	1	1	0	1	1	1	NC	0	0	1	0	1	0
Nargiso, 2013	1	NC	1	1	0	0	0	0	0	1	1	0	0
Perkins, 2014	1	1	1	1	1	1	1	0	0	1	1	1	0
Chang, 2014	1	1	0	0	1	1	0	0	0	1	1	0	0
Brown, 2015	1	1	1	1	1	1	0	0	1	1	1	1	0
Bornstein, 2015	1	1	0	1	1	1	0	1	1	1	0	1	0
Oetzel, 2015	1	1	0	0	1	1	1	1	1	1	0	1	0
Oetzel, 2015^b^	1	1	1	1	1	1	0	0	1	1	1	0	0
Stocks, 2015	1	NC	1	0	1	0	0	0	0	1	1	0	0
Brown, 2016	1	1	0	1	1	1	0	0	1	1	1	1	0
Goodman, 2017	1	1	0	1	1	NC	0	0	0	1	1	0	0
Okazaki, 2017	1	1	1	1	0	1	0	0	0	1	1	0	1
Jones, 2018	1	1	0	1	1	1	0	0	0	1	1	1	0
West, 2018	1	1	0	0	1	1	0	1	0	1	1	1	1
Oetzel, 2018	1	1	1	1	1	1	0	0	1	1	1	1	0
Duran, 2019	1	1	0	1	1	1	0	1	1	1	1	1	0
Soobiah, 2019	1	1	0	0	1	1	0	0	0	1	1	1	0
Dickson, 2020	1	1	0	1	1	1	0	0	NC	1	0	1	0
Rodriguez Espinosa, 2020	1	1	1	1	1	1	0	0	0	1	0	0	0
Lucero, 2020	1	1	1	1	1	1	0	0	1	1	1	1	0
Van Schelven, 2020	1	1	1	0	1	1	1	0	0	1	0	1	0
Hamilton, 2021	1	1	1	0	1	1	1	NC	NC	1	1	1	0
Boursaw, 2021	1	1	1	1	1	1	NC	0	1	1	1	1	0
Loban, 2021	1	1	0	0	1	1	1	1	1	1	0	1	0

First author, year	Q14	Q15	Q16	Q17	Q18	Q19	Q20	D1	D2	D3	D4	%^a^	
Butterfoss, 1996	1	1	1	1	0	0	1	3	1	6	1	55	
Kegler, 1998	0	1	1	1	0	0	1	2	1	4	1	40	
Chan, 2000	NC	NC	0	1	0	0	1	4	2	4	1	55	
Shortell, 2002	NC	0	0	1	0	0	1	4	0	4	1	45	
Weiss, 2002	NC	0	1	1	NC	1	1	4	2	5	2	65	
El Ansari, 2004	1	0	0	1	0	1	0	4	0	5	1	50	
El Ansari, 2004b	1	1	1	1	0	0	1	2	2	6	1	55	
Metzger, 2005	1	NC	0	1	0	0	1	2	2	6	1	55	
Kegler, 2005	1	1	1	1	NC	0	1	3	2	6	1	60	
Cramer, 2006	0	0	0	1	0	1	1	2	1	4	2	45	
Feinberg, 2008	1	NC	0	1	0	NC	1	3	2	5	1	55	
Feinberg, 2008b	1	1	0	0	NC	1	1	4	1	6	2	65	
Orr Brawer, 2008	1	1	1	1	1	1	1	4	2	7	3	80	
King, 2009	NC	0	0	1	1	NC	1	3	2	4	2	55	
Tolma, 2009	NC	0	0	1	NC	1	1	1	0	2	2	25	
Wagemakers, 2010	0	NC	1	1	0	NC	1	2	3	5	1	55	
King, 2010	1	1	1	1	1	0	1	3	2	7	2	70	
Ziff, 2010	0	NC	0	1	0	0	1	3	2	3	1	45	
Jones, 2011	NC	NC	0	NC	1	0	1	4	1	3	2	50	
Jones, 2011b	0	1	0	1	1	0	1	3	2	5	2	60	
Perkins, 2011	NC	1	1	1	1	1	1	4	2	6	3	75	
Bilodeau, 2011 and 2019^b^	NC	NC	0	1	1	1	1	3	1	4	3	55	
Curro, 2012	0	1	1	0	0	0	1	2	2	3	1	40	
El Ansari, 2012	0	0	1	1	1	0	0	3	2	4	1	50	
Brown, 2012	0	1	0	1	0	0	1	3	2	4	1	50	
Nargiso, 2013	1	NC	0	1	NC	NC	0	3	0	4	0	35	
Perkins, 2014	1	0	1	1	1	0	0	4	3	6	1	70	
Chang, 2014	0	NC	1	0	NC	NC	1	2	2	3	1	40	
Brown, 2015	0	1	1	1	0	0	1	4	2	7	1	70	
Bornstein, 2015	1	0	1	1	NC	0	1	3	2	7	1	65	
Oetzel, 2015	0	NC	0	1	1	0	1	2	3	5	2	60	
Oetzel, 2015^b^	1	NC	1	1	1	NC	1	4	2	6	2	70	
Stocks, 2015	1	1	0	1	1	1	1	2	1	5	3	55	
Brown, 2016	1	0	1	1	0	0	1	3	2	7	1	65	
Goodman, 2017	NC	0	1	1	0	NC	1	3	1	4	1	45	
Okazaki, 2017	1	1	1	1	1	1	0	4	1	7	2	70	
Jones, 2018	0	NC	0	1	0	0	1	3	2	4	1	50	
West, 2018	1	1	1	1	1	0	1	2	2	9	2	75	
Oetzel, 2018	1	1	1	1	1	NC	1	4	2	8	2	80	
Duran, 2019	NC	1	1	1	1	0	1	3	2	8	2	75	
Soobiah, 2019	0	0	1	1	NC	0	1	2	2	5	1	50	
Dickson, 2020	1	1	1	1	NC	0	1	3	2	6	1	60	
Rodriguez Espinosa, 2020	NC	NC	0	1	1	1	1	4	2	2	3	55	
Lucero, 2020	0	0	1	1	NC	0	1	4	2	6	1	65	
Van Schelven, 2020	1	NC	1	1	NC	0	1	3	3	5	1	60	
Hamilton, 2021	1	NC	1	1	1	0	1	3	3	6	2	70	
Boursaw, 2021	1	NC	1	1	NC	0	1	4	2	7	1	70	
Loban, 2021	1	1	1	1	1	NC	1	2	3	8	2	75	
All studies							Mean SD	3.04 0.82	1.77 0.78	5.27 1.62	1.52 0.71	58.02 12.32	

Q-SSP questions: Q1 Problem or phenomenon defined, described and justified? Q2 Population defined, described and justified? Q3 Specific research questions and/or hypotheses stated? Q4 Operational definitions of all study variables provided? Q5 Participant* inclusion criteria stated? Q6 Participant* recruitment strategy described? Q7 Justification/rationale for the sample size provided? Q8 Attrition* rate provided? Q9 Method of treating attrition* provided? Q10 Data analysis techniques justified—link between hypotheses, aims, research questions and analyses explained? Q11 Tool and/or all measures provided in report or supplement? Q12 Evidence for validity of measures present? Q13 Characteristics of person(s) collecting data (e.g. training, expertise, other demographic characteristics) provided? Q14 Information about context of data collection provided? Q15 Information about duration of data collection provided? Q16 Description of key demographic characteristics of study sample* provided? Q17 Discussion confined to the population* from which the sample was drawn? Q18 Informed consent or assent requested? Q19 Participants* debriefed at the end of data collection? Q20 Funding sources and conflicts of interest disclosed? [*questions adapted to reflect individual, project and/or partnership based on study unit of analysis]

Scoring

D1 Domain 1: introduction/rationale/variables score (ratio of sum Q1–Q4/total applicable domain items)

D2 Domain 2: participants*/sampling/recruitment score (ratio of sum Q5–Q7/total applicable domain items)

D3 Domain 3: data collection/analyses/measures/results/discussion score (ratio of sum Q8–17/total applicable domain items)

D4 Domain 4: ethics score (ratio of sum Q18–Q20/total applicable domain items)

^a^Total overall quality score (%) (sum D1–D4/total applicable domain items × 100)

^b^Companion report

Across studies, the *Introduction* domain mean score was 3.04/4.00 points (SD0.82), the *Participant* domain mean score was 1.77/3.00 points (SD0.78), the *Data* domain mean score was 5.27/10.00 points (SD1.62), and the *Ethics* domain mean score was 1.52/3.00 points (SD0.71).

The problem and target population were generally well described and participant sampling and recruitment details present, but operational definitions (32, 67%), research questions and hypotheses (24, 50%) and sample size justification were often lacking (35, 75%). There were strong links between the proposed and presented analyses (46, 96%), but the study measures themselves were frequently missing from reports or supplements (17, 35%). The provision of validity evidence for included measures was found lacking in almost a third of studies (14, 29%), and most studies lacked detail about those collecting data (42, 88%), the duration of data collection (29, 60%) and the study context (25, 52%). Explicit reference to informed consent/assent and the inclusion of participants in post-data-collection debriefing was largely absent or unclear across included studies (29, 60% and 37, 77%, respectively).

Overall, four of the six studies with “acceptable” quality overlapped with studies reporting more comprehensive psychometrics [61, 65, 71, 88], but only two overlapped with those reporting higher pragmatic tool criteria scores [61, 71].

### Evidence summary: tool validity, reliability, pragmatics and study quality

This review identified 58 tools underpinned by empirical psychometric evidence in the assessment of health research partnership outcomes and impacts. When considered with pragmatic tool evaluation criteria and study quality score findings, four noteworthy groups of studies and accompanying tools emerged (22, 46%). First, only two studies (2, 4%) reported more comprehensive psychometrics and had both high pragmatic tool criteria and Q-SSP study quality scores [61, 71]. A second group of studies (7, 15%) reported more comprehensive psychometrics and either high pragmatic tool criteria scores [68, 69, 72, 74, 80] or high study quality scores [65, 88]. The third group (8, 17%) had more comprehensive psychometrics [60, 78, 79, 82–85, 87], and the last set of studies (5 plus companion report, 10%) scored high on pragmatic tool evaluation criteria [62–64, 67, 70, 73].

## Discussion

This systematic review identified 58 tools for assessing health research partnership outcomes and impacts with tool psychometric evidence and pragmatic characteristics. We were able to identify a group of noteworthy tools, distinguished by their psychometric evidence, tool pragmatic characteristics and study quality scores.

### Key study-level comparative findings

Overall, the presence and reporting of empirical psychometric evidence and pragmatic characteristics appeared improved in our study compared with previous reviews, yet several challenges related to the nascency of this research field remain (e.g. lack of key term definitions and measurement clarity, term switching, a lack of studies with deliberate focus on tool development, testing, evaluation and improvement, variable and inconsistent reporting). Future research to advance partnership measurement and science should consider both psychometric improvements (with specific emphasis on increased consistency, level of tested and reported detail, and dedicated study) and pragmatic considerations (specifically on accessible tools that are better informed by partner experiences and expertise, designed for partnership monitoring, and quantifiably readable). In examining tools with empirical psychometric evidence, this study contributes to our understanding of existing partnership tool measurement strengths and gaps. Our review provides practical ways to advance partnership measurement and, ultimately, partnership science.

At the study level, our findings aligned with previous reviews in that most included studies were North American- and English-centric, with a wide publication dispersion pattern and mid-2010 emergence [2, 7, 8, 11]. We also experienced previously reported challenges in the location of tools and author responsiveness [5, 7]. Our study differed from others documenting a predominance of qualitative methods and relative rarity of quantitative tools, designs and methods [9, 12, 70, 90–92]. By contrast, our review deliberately sought and identified tools with empirical psychometric and pragmatic characteristics encompassing diverse health research approaches. This review identified studies employing cross-sectional and mixed-method/embedded survey designs and quantitative and mixed methods; this catchment is likely a function of our study inclusion criteria but may also reflect an increasing overall trend towards the quantification of partnership assessment [1, 7, 11–13, 92, 93].

### Key tool-level comparative findings

On a tool level, we found similarities and differences between our study and previous, related reviews, but these studies differed in scope (e.g. literature, search period, research domains other than health, focus of measurement) and definitions of *partnership,* generating very different samples and eligible primary literature [2].

Our findings demonstrate the need for research deliberately focused on tool development, testing and evaluation. Like other related health research partnership reviews [7, 8, 10, 94], we found that while tool purpose was universally reported, investigators focused almost exclusively on assessing and understanding the characteristics of bespoke partnerships. This was a consistent finding, despite the diverse scope and focus of these reviews (i.e. patient/public evaluation tools, community coalitions, coproduction impacts, and research collaboration quality and outcomes, respectively). Very few primary studies in our review focused specifically on tool validation or psychometric testing, although most involved one or more such activities. Furthermore, most studies were multifocal, that is, encompassing one or more tool development, modification, use, evaluation or validation activities simultaneously. These findings support previous reports regarding the paucity of focused health research partnership tool evaluation research [10, 94]. Our findings strengthen existing recommendations targeting the systematic assessment of psychometric and pragmatic tool properties [8], and more deliberate funding of research on tool design, testing, improvement and evolvement in general [49]. These aspects are considered key to advancing partnership science measurement and partnership science as a field [8, 9, 70, 95].

Conceptually, our study revealed a much higher presence of theoretical underpinnings at both the study and tool levels (91%, respectively), compared with levels reported in other partnership tool reviews of patient/public and community coalition evaluation tools [7, 94]. However, the implications of this finding remain unclear. Some authors have observed that theoretical/conceptual connections to both partnership and measurement theory rarely translate into operationalized tool elements [8, 17]; this is an important area of future inquiry.

The tools we reviewed measured outcomes similarly, as compared with a recent review of patient/public partnership evaluation tools (52% vs 56%) [7]; however, in our study, we found that explicit definitions for outcome and impact terms were present intermittently and often interchanged. Terminology challenges have been reported in other systematic studies in the health research partnerships domain, noting the significant variance, overlap and omission of key term definitions from reports (i.e. terms for outcomes/impacts, partnership approaches and tool types) [9, 14, 15, 96]. While comparative research and crosstalk among research partnership traditions is a relatively recent phenomenon [4, 6, 96–99], clarity on key concepts, terminology, definitions, core measures and tools is fundamental to advancing partnership measurement and scientific inquiry [8, 9, 49, 70].

### Comparative findings: tool pragmatic characteristics, validity and reliability

Pragmatic tool evaluation scores were generally higher in our review than in Boivin and colleagues’ review of patient partnership evaluation tools [7]. In our study, the highest mean domain scores were *Comprehensiveness* and *Scientific Rigour*, whereas *Scientific Rigour* was the lowest domain score in the Boivin review [7]). Importantly, we found that only a single tool overlapped between the reviews. This lack of overlap can be accounted for by differences in review scope, targets and inclusion criteria (i.e. the Boivin review focused on patient and public involvement evaluation tools and included tools for assessing engagement in both health system decision-making and health research, with narrower search terms over a shorter time span; and our review deliberately selected studies reporting empirical tool validity and reliability evidence).

Tool validity (86%) and reliability (95%) evidence in our study was markedly higher and contrasted starkly with prior work [7, 8], in which evidence for validity was found in only 48% and 7% of studies, respectively [7, 8], and evidence for reliability was found in 45% and 35% of studies, respectively [7, 8]. As noted previously, there was little to no overlap in captured tools between these reviews (*n* = 1 [7] and *n* = 13 [8], respectively), which can be similarly accounted for by differences in scope that generated different primary and secondary literature sets. The MacGregor overview of reviews [8] focused solely on reviews of tools to assess the impacts of research coproduction, differing by time span, key partnership terminology and key domains. As a result, only four of the eight identified reviews were considered in-scope; thus, the number of overlapping tools was limited (*n* = 13).

### Future research

Boateng et al. [49] describe the requisite steps, activities and key precursors and concurrent factors required for robust tool development, testing and evaluation in the future. Specific attention to such steps and components could enable more deliberate tool evolvement in the health research partnership assessment domain. Specifically, the authors call for graduate-level training in the development and evaluation of tools, to create expertise in graduate students and research teams. Furthermore, the authors caution that this research can be “onerous, jargon-filled, unfamiliar, and resource intensive” (p. 1) [49]. Specific accommodations to offset resource and time intensity and higher participant burden due to larger sample sizes may be required. Health research partnerships assessments must meet the needs of both researchers and end-users by balancing rigour and resource intensity in a way that remains fit for purpose. Both deliberate funding and the use of hybrid study designs will be helpful for providing required focus and generating robust evidence that will address persistent psychometric and pragmatic gaps with future research.

### Study limitations

We noted several key limitations with this review. We observed several challenges with respect to the evidence for and the testing of tool psychometric properties. Like Sandoval et al. [5], we experienced challenges related to the reporting of psychometrics on multiple levels (e.g. scale, index, subscale, item and tool), as well as mismatched use of psychometric evidence (e.g. justification or application of previous scale, subscale or item-specific psychometrics to other levels of testing). To mitigate this risk, we approached psychometric evidence in eligible studies with these issues in mind, and relied on strict methodological processes (independent, duplicate abstraction and review and resolution of all discrepancies through consensus discussions) to ensure accurate interpretation and representation of abstracted data.

As mentioned previously, the variable use of terminology may have compromised our ability to clearly describe and assess health research partnership tools. Further efforts to consolidate terms and definitions across health research partnership traditions will help resolve these issues in future work.

This study was limited in several ways by the accessibility and reporting concerns documented in previous reviews [3, 5, 7, 14, 15]. Most included studies were multimodal and did not often explicitly refer to tool development, testing or evaluation in their purpose statements. To mitigate the risk of missing potentially relevant studies in our review, we deliberately kept our inclusion criteria broad at the title and abstract (L1) screening phase. However, this strategy also produced a large set of L2 full-text assessments, negatively impacting study feasibility. Consensus and consolidation of evidence in this research domain, as well as more focused, explicit reporting of health research partnership assessment, tools and psychometric and pragmatic characteristics, will facilitate more efficient literature location, retrieval and assessment in the future.

Finally, we noted a potential gap in the scope of a question modified as part of the pragmatic tool evaluation criteria: *Was the tool informed by literature generated from a systematic literature search?* In retrospect, we surmise that this question was too narrow to capture evidence derived from historical hypothesis testing generated by theoretically driven research (i.e. dimensionality tests) [49]. In addition to synthesis-level evidence for relevant components, tools or tool components that are informed by iterative tests of components derived from conceptual framework testing could play an equal or more important role in identifying and refining key tool constructs. Theoretically grounded components may also progressively improve the psychometric quality of health research partnership outcome and impact assessment tools. We recommend amending this question for use in future tool evaluation studies to better capture the full scope of relevant evidence underlying assessment tools.

## Conclusions

This large-volume systematic review successfully identified empirically evidenced tools for the assessment of health research partnership outcomes and impacts. Our findings signal some promising improvements in the presence of conceptual, methodological and psychometric characteristics in measurement tools, and the availability of pragmatic tool characteristics. Persistent challenges linked to the nascency of the research partnership field and its measurement remain. Practically, the comprehensive tool characteristics presented here can help researchers and partners choose assessment tools that best fit their purposes and needs. Finally, our findings further strengthen calls for more deliberate and comprehensive tool development, testing, evaluation and reporting of psychometric and pragmatic characteristics to advance research partnership assessment and research partnership science domains.

Advancing knowledge of health research partnership outcomes and impacts assessment and partnership science are mandated aims of the IKTRN [100]. The IKTRN is a research network based at the Centre for Practice-Changing Research at the Ottawa Hospital and supported by the Canadian Institutes of Health Research. The IKTRN comprises researchers from more than 30 universities and research centres and research users from over 20 organizations, with a broad research agenda focused on best practices and their routine application to ensure effective, efficient and appropriate healthcare [101, 102].

## Supplementary Information

**Additional file 1: Appendix S1.** Systematic review protocol deviations and rationale. **Appendix S2.** Glossary of terms. **Appendix S3.** Translated search strategy. **Appendix S4.** Health research partnership pragmatic tool evaluation criteria. **Appendix S5.** Quality assessment checklist for survey studies in psychology (Q-SSP) criteria. **Appendix S6.** Bibliography of included studies. **Appendix S7.** PRISMA-systematic review checklist.

## Data Availability

The study search strategy, abstraction tools and bibliographic tool index will be available through the Open Science Framework upon completion of the research and publication of findings. Data generated and/or analysed during the current study will be made available upon reasonable request, after completion of the dissertation research and publication of findings, from the first author.
