# Supplementary material for: Tools for assessing health research partnership outcomes and impacts: a systematic review
Source: Health Res Policy Syst. 2023 Jan 5;21:3. doi: 10.1186/s12961-022-00937-9 (PMC9817421; doi:10.1186/s12961-022-00937-9)
Supplement: Supplementary file 1 — Additional file 1: Appendix S1. Systematic review protocol deviations and rationale. Appendix S2. Glossary of terms. Appendix S3. Translated search strategy. Appendix S4. Health research partnership pragmatic tool evaluation criteria. Appendix S5. Quality assessment checklist for survey studies in psychology (Q-SSP) criteria. Appendix S6. Bibliography of included studies. Appendix S7. PRISMA-systematic review checklist. [file 12961_2022_937_MOESM1_ESM.docx]

Additional File 1

[**Appendix S1:** Systematic Review Protocol Deviations and Rationale 2](#_Toc96023783)

[**Appendix S2.** Glossary of Terms 4](#_Toc96023784)

[**Appendix S3.** Translated Search Strategy 6](#_Toc96023785)

[**Appendix S4.** Health Research Partnership Pragmatic Tool Evaluation Criteria 14](#_Toc96023786)

[**Appendix S5.** Quality Assessment Checklist for Survey Studies in Psychology (Q-SSP) Criteria 17](#_Toc96023787)

[**Appendix S6:** Bibliography of included studies 19](#_Toc96023788)

[**Appendix S7.** PRISMA-Systematic Review Checklist 22](#_Toc96023789)

[**References** 25](#_Toc96023790)

# **Appendix S1:** Systematic Review Protocol Deviations and Rationale

| **Protocol Deviation** | **Rationale** |
| --- | --- |
| **Electronic databases** | The original search included Scopus and Web of Science databases. After conducting the scout search, these databases were removed prior to PRESS review for sensitivity and feasibility reasons[1, 2]. |
| **Grey literature and referral literature** | We initially proposed retrieval of grey and referral literature, but subsequently removed these data sources. We found most scout search returns were replicated in the peer-reviewed literature and the benefit of these additional data sources was marginal, thus were removed. |
| **Duplicate, Independent Abstraction** | Data type (qualitative) and feasibility concerns drove our decision to alter the abstraction strategy. Independent duplicate abstraction was replaced by a hybrid strategy comprising independent abstraction with independent validation by a second, trained co-investigator. All discrepancies were discussed to consensus and resolution. |
| **Key Terms and Definitions** | We altered our definition of *tool* after observing how distinct the content of citations pertaining to indicators and metrics were. We removed these two terms and excluded the papers from the current review, however, flagged them to perform a separate examination and synthesis of this data subset in future.  We replaced the term ‘stakeholder’ with ‘partner’ throughout this review to ensure our approach is respectful, inclusive, and acknowledges and helps to reconcile historical harms[3].  The term ‘outcome’ was altered slightly to accommodate for positive and negative effects and process outcomes, as follows: ‘factor(s) described in the study methods used to determine a change in status as a result of interventions, can be measured or assessed as component(s) of the study, and are not futuristic’; including both process and summative outcomes. (Adapted from Hoekstra et al, 2018 and University of Waterloo, 2018)[4, 5]  The term ‘impact’ was altered to better reflect a range of potential impacts, as follows: ‘effects, influences or changes to the economy, society, public policy or services, individuals, teams, organizations, health, the environment, or quality of life, beyond academia’.(Adapted from Hoekstra et al, 2018 and Higher Education and Funding Council for England, 2014)[4, 6] |
| **Inclusion-Exclusion Criteria** | Indicators and metrics were specifically excluded from this review, as the nature and contents of these reports were distinct. These studies will be reviewed and analyzed for reporting in a separate, future publication. |
| **Pragmatic and Psychometrics Assessment Criteria** | The level and type of detail required to assess tools using the initially-proposed PAPERS criteria[7, 8] exceeded what was abstractable from the literature we retained. There were a high number of incomplete cells due to missing data. We replaced the PAPERS psychometric and pragmatic criteria [7] and the Global Assessment/Referral Criteria[9] with the newly modified tool evaluation criteria. We chose instead to modify a set of consensus-built evaluative criteria developed by the Centre of Excellence on Partnership with Patients and the Public[10] as part of the *Patient and Public Engagement Evaluation Toolkit Project.* These criteria were utilized by Boivin and colleagues in their 2018 systematic review [11].  The criteria were developed by- and for a diverse set of partners, including patients, members of the public, healthcare providers, decision makers, community members, and academic researchers. We consulted with A L’Esperance to craft and pilot-test a modified version of the CEPPP criteria for our study purposes. |
| **Outcomes Variables** | Descriptive study characteristics  [author, year, title, language, country, health sub-domain, focus (discipline), population targeted by tool, sex (if available), study design, study method(s), conceptual framework (study)] Reported Elsewhere: [Study outcomes and Impacts]  Tool Characteristics  [tool name, tool type, tool purpose, underlying theory/model/framework (tool), theory citation (tool), level of analysis, recommended frequency of use; Unavailable: [measurement scale type – not systematically reported]; Reported elsewhere: [tool constructs/categories, definitions]  Psychometric Criteria  [reported evidence of reliability (internal consistency, test-retest reliability, inter-rater reliability, reliability(other); reported evidence of validity (construct validity (convergent, factorial, discriminant, known groups, undefined); criterion validity (predictive, concurrent); content validity, structural validity (dimensionality); face validity, responsiveness); reported evidence of norms; reported evidence of interpretability (ceiling and/or floor effects, interpretability (other)]  Pragmatic Criteria  [Health research partnership tool assessment criteria (modified from Boivin et al, 2018 – see Appendix) replaced the PAPERS and global assessment criteria as described above; modified criteria were a better, more feasible fit with source data and level of reported detail]  Partnership Characteristics [reported elsewhere] |

# **Appendix S2.** Glossary of Terms

| **Key Term** | **Definition** |
| --- | --- |
| Health Research Partnership[4, 12] | “Partnerships involving individuals, groups, or organizations engaged in collaborative health research activity involving at least one researcher (e.g., an individual affiliated with an academic department, hospital or medical centre), and any partner actively engaged in any part of the research process (e.g., decision or policy maker, health care administrator or leader, community agency, charities, network, patients, industry partner, etc).”  A health research partnership may encompass a diverse set of research activities including (but not limited to) integrated KT (IKT), community based participatory research (CBPR), action research or participatory action research (PAR), collaborative research, co-design, academic-community partnerships and their derivatives. |
| Tool[4, 13] | “An instrument (survey, measures, assessments, questionnaire, inventory, checklist, list of factors, subscales or similar) that can be used to assess the outcome or impact elements or domains of a health research partnership.” |
| Outcome (Adapted from University of Waterloo, 2018)[4, 5] | “Factor(s) described in the study methods used to determine a change in status as a result of interventions, can be measured or assessed as component(s) of the study, and are not futuristic”; including both process and summative outcomes. |
| Impact (Adapted from Hoekstra et al, 2018 and the Higher Education Funding Council for England, 2014) [4, 6] | “…effects, influences or changes to the economy, society, public policy or services, individuals, teams, organizations, health, the environment, or quality of life, beyond academia” |
| Context[4, 14] | “The physical, organizational, institutional, and legislative structures that enable and constrain, and resource and realize, people and procedures.” |
| **Psychometric Terms (Reliability)** | |
| Internal consistency | A measure of the extent to which items in a tool are intercorrelated. Internal consistency assesses the extent to which items measure the same concept and tested using Cronbach’s alpha (Adapted from Terwee et al, 2007)[15]. |
| Test-retest reliability | An assessment of the degree to which participants’ performance is consistent on tests of the same construct at two different time points. (Adapted from Bhattacherjee, 2012)[16] |
| Inter-rater reliability | An assessment of the extent to which scores measuring the same construct are consistent when assessed by two or more independent raters. (Adapted from Kimel & Revicki, 2014)[17] |
| **Psychometric Terms (Validity)** | |
| Content Validity | The extent to which the a set of tool items matches the content domain of the construct the items are intended to measure (Adapted from Bhattacherjee, 2012)[16] |
| Face Validity | The degree to which an item is a reasonable measure of an underlying construct. (Adapted from Bhattacherjee, 2012)[16] |
| Construct Validity | A measure of the extent to which tool scores relate in a manner that is consistent with theoretical hypotheses, related to the measures under study. (Adapted from Terwee et al, 2007)[15] |
| 1. Convergent Validity | An assessment of the degree of proximity between measures and the construct they intend to measure. (Adapted from Bhattacherjee, 2012)[16] |
| 1. Discriminant Validity | An assessment of the degree to which a measure can discriminate between constructs it is intended and not intended to measure. (Adapted from Bhattacherjee, 2012)[16] |
| 1. Known Groups | The extent to which the tool under study can discriminate between participants known to possess a trait compared to those who do not possess the same trait. (Adapted from Davidson, 2014)[18] |
| 1. Factorial Validity | An assessment of the correlation of responses for a group of tool items. (Adapted from Piedmont, 2014)[19] |
| Criterion Validity | A measure of how well a chosen measure relates to external criterion using empirical observations. (Adapted from Bhattacherjee, 2012)[16] |
| 1. Predictive Validity | An assessment to determine whether scores predict future outcomes that they are hypothesized to predict. (Adapted from Boateng et al, 2018)[20] |
| 1. Concurrent Validity | An assessment of how well a new tool compares to an established tool, or alternatively, the concurrent assessment of two groups at the same time or two different groups being tested simultaneously. (Adapted from Boateng et al, 2018)[20] |
| Dimensionality (Structural Validity) | An assessment of whether the hypothesized structure fits the items under scrutiny. (Adapted from Boateng et al, 2018)[20] |
| Responsiveness | The ability of a tool to detect change over time when a change is known to have occurred. (Adapted from Terwee et al, 2007)[15] |
| Norms | Norms are standards against which individual scores can be compared (norm groups can be defined by gender, race, ethnicity, socio-economic status, education or other norm-defined characteristics). (Adapted from Chien & Yao, 2014)[21] |
| Interpretability | The degree to which one can assign qualitative meaning to quantitative scores. (Adapted from Terwee et al, 2007)[15] |
| 1. Floor and Ceiling Effects | The number of respondents who achieved the lowest or highest possible score. (Adapted from Terwee et al, 2007)[15] |

# **Appendix S3.** Translated Search Strategy

**Source strategy; Ovid MEDLINE**

| Database: "Ovid MEDLINE(R) Epub Ahead of Print, In-Process & Other Non-Indexed Citations, Ovid MEDLINE(R) Daily and Ovid MEDLINE(R) 1946 to Present” | |
| --- | --- |
| Line | Search terms |
| 1 | ("community partners" adj2 (research* or engage*)).ab,ti |
| 2 | ("as partners" adj4 research).ab,ti |
| 3 | ("community engagement" AND research).ab,ti |
| 4 | ("community involvement" AND research).ab,ti |
| 5 | ("community participation" adj3 research).ab,ti |
| 6 | ("knowledge users" adj3 research).ab,ti |
| 7 | ("service user" AND (involvement OR engagement)).ab,ti |
| 8 | ((clinical or health) adj4 partnership*).ab,ti |
| 9 | (collaborat* adj3 (partner* or research*)).ab,ti |
| 10 | ((engag* or participation) adj2 stakeholder?).ab,ti |
| 11 | ((involvement or engagement) adj4 research).ab,ti |
| 12 | (partnership* adj4 research*).ab,ti |
| 13 | (action research).ab,ti |
| 14 | (coalition? adj3 health).ab,ti |
| 15 | (cocreation).ab,ti |
| 16 | (co-creation).ab,ti |
| 17 | (community coalition?).ab,ti |
| 18 | (community-based research).ab,ti |
| 19 | (community-engaged research).ab,ti |
| 20 | (consumer involvement).ab,ti |
| 21 | (coproduction AND research).ab,ti |
| 22 | (co-production AND research).ab,ti |
| 23 | (coresearch*).ab,ti |
| 24 | (co-research*).ab,ti |
| 25 | (disseminat* research).ab,ti |
| 26 | (emancipatory research).ab,ti |
| 27 | (engage* adj3 research*).ab,ti |
| 28 | (engaged scholarship).ab,ti |
| 29 | (inclusive adj2 research).ab,ti |
| 30 | ("knowledge transfer and exchange").ab,ti |
| 31 | (knowledge translation).ab,ti |
| 32 | (participatory design).ab,ti |
| 33 | (participatory evaluation).ab,ti |
| 34 | (participatory intervention?).ab,ti |
| 35 | (participatory research).ab,ti |
| 36 | ("patient and public involvement").ab,ti |
| 37 | (patient-centered adj2 research).ab,ti |
| 38 | (peer research).ab,ti |
| 39 | (research adj2 translation).ab,ti |
| 40 | (("mode 2" or "mode two" or "mode II") adj3 (knowledge or research)).ab,ti |
| 41 | ("linkage and exchange").ab,ti |
| 42 | (research and ("peer led" or "public led" or "patient led" or "stakeholder led")).ab,ti |
| 43 | 1 or 2 or 3 or 4 or 5 or 6 or 7 or 8 or 9 or 10 or 11 or 12 or 13 or 14 or 15 or 16 or 17 or 18 or 19 or 20 or 21 or 22 or 23 or 24 or 25 or 26 or 27 or 28 or 29 or 30 or 31 or 32 or 33 or 34 or 35 or 36 or 37 or 38 or 39 or 40 or 41 or 42 |
| 44 | (community-based participatory research).kw,kf |
| 45 | (participatory research).kw,kf |
| 46 | (integrated knowledge translation).kw,kf |
| 47 | (action research).kw,kf |
| 48 | (community involvement).kw,kf |
| 49 | (participatory action research).kw,kf |
| 50 | ("patient and public involvement").kw,kf |
| 51 | (cocreation).kw,kf |
| 52 | (co-creation).kw,kf |
| 53 | (Collaborative research).kw,kf |
| 54 | (Community engagement).kw,kf |
| 55 | (community-academic partnership).kw,kf |
| 56 | (community-based research).kw,kf |
| 57 | (coproduction).kw,kf |
| 58 | (co-production).kw,kf |
| 59 | (Inclusive research).kw,kf |
| 60 | (knowledge exchange).kw,kf |
| 61 | (participatory research partnership?).kw,kf |
| 62 | (partnership research).kw,kf |
| 63 | (Patient participation).kw,kf |
| 64 | (researcher-stakeholder collaboration).kw,kf |
| 65 | 44 or 45 or 46 or 47 or 48 or 49 or 50 or 51 or 52 or 53 or 54 or 55 or 56 or 57 or 58 or 59 or 60 or 61 or 62 or 63 or 64 |
| 66 | 43 or 65 |
| 67 | ((outcome? OR impact?) AND (measur* OR assess* OR evaluat*)).ab,ti |
| 68 | ((outcome? or impact?) AND (tool? OR instrument?)).ab,ti |
| 69 | ((measur* OR assess* OR evaluat*) AND (tool? OR instrument?)).ab,ti |
| 70 | 67 or 68 or 69 |
| 71 | Benchmarking/ |
| 72 | "Surveys and Questionnaires"/ |
| 73 | Program Evaluation/ |
| 74 | "Outcome Assessment (Health Care)"/ |
| 75 | "Outcome and Process Assessment (Health Care)"/ |
| 76 | "Process Assessment (Health Care)"/ |
| 77 | Health Impact Assessment/ |
| 78 | Reproducibility of Results/ |
| 79 | Psychometrics/ |
| 80 | 71 or 72 or 73 or 74 or 75 or 76 or 77 or 78 or 79 |
| 81 | 70 or 80 |
| 82 | 65 and 81 |

#### **CINAHL Translation**

TI ((("community partners" N2 (research* or engage*)) OR ("as partners" N4 research) OR ("community engagement" AND research) OR ("community involvement" AND research) OR ("community participation" N3 research) OR ("knowledge users" N3 research) OR ("service user" AND (involvement OR engagement)) OR ((clinical or health) N4 partnership*) OR (collaborat* N3 (partner* or research*)) OR ((engag* or participation) N2 stakeholder#) OR ((involvement or engagement) N4 research) OR (partnership* N4 research*) OR ("action research") OR (coalition# N3 health) OR (cocreation) OR (co-creation) OR ("community coalition#") OR ("community-based research") OR ("community-engaged research") OR ("consumer involvement") OR (coproduction AND research) OR (co-production AND research) OR (coresearch*) OR (co-research*) OR ("disseminat* research") OR ("emancipatory research") OR (engage* N3 research*) OR ("engaged scholarship") OR (inclusive N2 research) OR ("knowledge transfer and exchange") OR ("knowledge translation") OR ("participatory design") OR ("participatory evaluation") OR ("participatory intervention#") OR ("participatory research") OR ("patient and public involvement") OR (patient-centered N2 research) OR ("peer research") OR (research N2 translation) OR (("mode 2" or "mode two" or "mode II") N3 (knowledge or research)) OR ("linkage and exchange") OR (research and ("peer led" or "public led" or "patient led" or "stakeholder led"))) ) OR AB ( (("community partners" N2 (research* or engage*)) OR ("as partners" N4 research) OR ("community engagement" AND research) OR ("community involvement" AND research) OR ("community participation" N3 research) OR ("knowledge users" N3 research) OR ("service user" AND (involvement OR engagement)) OR ((clinical or health) N4 partnership*) OR (collaborat* N3 (partner* or research*)) OR ((engag* or participation) N2 stakeholder#) OR ((involvement or engagement) N4 research) OR (partnership* N4 research*) OR ("action research") OR (coalition# N3 health) OR (cocreation) OR (co-creation) OR ("community coalition#") OR ("community-based research") OR ("community-engaged research") OR ("consumer involvement") OR (coproduction AND research) OR (co-production AND research) OR (coresearch*) OR (co-research*) OR ("disseminat* research") OR ("emancipatory research") OR (engage* N3 research*) OR ("engaged scholarship") OR (inclusive N2 research) OR ("knowledge transfer and exchange") OR ("knowledge translation") OR ("participatory design") OR ("participatory evaluation") OR ("participatory intervention#") OR ("participatory research") OR ("patient and public involvement") OR (patient-centered N2 research) OR ("peer research") OR (research N2 translation) OR (("mode 2" or "mode two" or "mode II") N3 (knowledge or research)) OR ("linkage and exchange") OR (research and ("peer led" or "public led" or "patient led" or "stakeholder led"))))

AND

(MH "Surveys") OR (MH "Program Evaluation") OR (MH "Benchmarking") OR (MH "Process Assessment (Health Care)") OR (MH "Outcome Assessment") OR (MH "Health Impact Assessment") OR (MH "Reproducibility of Results") OR (MH "Psychometrics") OR TI ( (((outcome# OR impact#) AND (measur* OR assess* OR evaluat*)) OR ((outcome# or impact#) AND (tool# OR instrument#)) OR ((measur* OR assess* OR evaluat*) AND (tool# OR instrument#))) ) OR AB ( (((outcome# OR impact#) AND (measur* OR assess* OR evaluat*)) OR ((outcome# or impact#) AND (tool# OR instrument#)) OR ((measur* OR assess* OR evaluat*) AND (tool# OR instrument#))) )

#### **PsycInfo Translation**

TI ((("community partners" N2 (research* or engage*)) OR ("as partners" N4 research) OR ("community engagement" AND research) OR ("community involvement" AND research) OR ("community participation" N3 research) OR ("knowledge users" N3 research) OR ("service user" AND (involvement OR engagement)) OR ((clinical or health) N4 partnership*) OR (collaborat* N3 (partner* or research*)) OR ((engag* or participation) N2 stakeholder#) OR ((involvement or engagement) N4 research) OR (partnership* N4 research*) OR ("action research") OR (coalition# N3 health) OR (cocreation) OR (co-creation) OR ("community coalition#") OR ("community-based research") OR ("community-engaged research") OR ("consumer involvement") OR (coproduction AND research) OR (co-production AND research) OR (coresearch*) OR (co-research*) OR ("disseminat* research") OR ("emancipatory research") OR (engage* N3 research*) OR ("engaged scholarship") OR (inclusive N2 research) OR ("knowledge transfer and exchange") OR ("knowledge translation") OR ("participatory design") OR ("participatory evaluation") OR ("participatory intervention#") OR ("participatory research") OR ("patient and public involvement") OR (patient-centered N2 research) OR ("peer research") OR (research N2 translation) OR (("mode 2" or "mode two" or "mode II") N3 (knowledge or research)) OR ("linkage and exchange") OR (research and ("peer led" or "public led" or "patient led" or "stakeholder led"))) ) OR AB ( (("community partners" N2 (research* or engage*)) OR ("as partners" N4 research) OR ("community engagement" AND research) OR ("community involvement" AND research) OR ("community participation" N3 research) OR ("knowledge users" N3 research) OR ("service user" AND (involvement OR engagement)) OR ((clinical or health) N4 partnership*) OR (collaborat* N3 (partner* or research*)) OR ((engag* or participation) N2 stakeholder#) OR ((involvement or engagement) N4 research) OR (partnership* N4 research*) OR ("action research") OR (coalition# N3 health) OR (cocreation) OR (co-creation) OR ("community coalition#") OR ("community-based research") OR ("community-engaged research") OR ("consumer involvement") OR (coproduction AND research) OR (co-production AND research) OR (coresearch*) OR (co-research*) OR ("disseminat* research") OR ("emancipatory research") OR (engage* N3 research*) OR ("engaged scholarship") OR (inclusive N2 research) OR ("knowledge transfer and exchange") OR ("knowledge translation") OR ("participatory design") OR ("participatory evaluation") OR ("participatory intervention#") OR ("participatory research") OR ("patient and public involvement") OR (patient-centered N2 research) OR ("peer research") OR (research N2 translation) OR (("mode 2" or "mode two" or "mode II") N3 (knowledge or research)) OR ("linkage and exchange") OR (research and ("peer led" or "public led" or "patient led" or "stakeholder led"))))

AND

TI ( (((outcome# OR impact#) AND (measur* OR assess* OR evaluat*)) OR ((outcome# or impact#) AND (tool# OR instrument#)) OR ((measur* OR assess* OR evaluat*) AND (tool# OR instrument#))) ) OR AB ( (((outcome# OR impact#) AND (measur* OR assess* OR evaluat*)) OR ((outcome# or impact#) AND (tool# OR instrument#)) OR ((measur* OR assess* OR evaluat*) AND (tool# OR instrument#))) )

#### **EMBASE Translation**

("community partners" adj2 (research* or engage*)).ab,ti.
("as partners" adj4 research).ab,ti.
("community engagement" and research).ab,ti.
("community involvement" and research).ab,ti.
("community participation" adj3 research).ab,ti.
("knowledge users" adj3 research).ab,ti.
("service user" and (involvement or engagement)).ab,ti.
((clinical or health) adj4 partnership*).ab,ti.
(collaborat* adj3 (partner* or research*)).ab,ti.
((engag* or participation) adj2 stakeholder?).ab,ti.
((involvement or engagement) adj4 research).ab,ti.
(partnership* adj4 research*).ab,ti.
action research.ab,ti.
(coalition? adj3 health).ab,ti.
cocreation.ab,ti.
co-creation.ab,ti.
community coalition?.ab,ti.
community-based research.ab,ti.
community-engaged research.ab,ti.
consumer involvement.ab,ti.
(coproduction and research).ab,ti.
(co-production and research).ab,ti.
coresearch*.ab,ti.
co-research*.ab,ti.
disseminat* research.ab,ti.
emancipatory research.ab,ti.
(engage* adj3 research*).ab,ti.
engaged scholarship.ab,ti.
(inclusive adj2 research).ab,ti.
"knowledge transfer and exchange".ab,ti.
knowledge translation.ab,ti.
participatory design.ab,ti.
participatory evaluation.ab,ti.
participatory intervention?.ab,ti.
participatory research.ab,ti.
"patient and public involvement".ab,ti.
(patient-centered adj2 research).ab,ti.
peer research.ab,ti.
(research adj2 translation).ab,ti.
(("mode 2" or "mode two" or "mode II") adj3 (knowledge or research)).ab,ti.
"linkage and exchange".ab,ti.
(research and ("peer led" or "public led" or "patient led" or "stakeholder led")).ab,ti.
or/1-42
community-based participatory [research.kw](http://research.kw/).
participatory [research.kw](http://research.kw/).
integrated knowledge [translation.kw](http://translation.kw/).
action [research.kw](http://research.kw/).
community [involvement.kw](http://involvement.kw/).
participatory action [research.kw](http://research.kw/).
"patient and public involvement".kw.
[cocreation.kw](http://cocreation.kw/).
[co-creation.kw](http://co-creation.kw/).
Collaborative [research.kw](http://research.kw/).
Community [engagement.kw](http://engagement.kw/).
community-academic [partnership.kw](http://partnership.kw/).
community-based [research.kw](http://research.kw/).
[coproduction.kw](http://coproduction.kw/).
[co-production.kw](http://co-production.kw/).
Inclusive [research.kw](http://research.kw/).
knowledge [exchange.kw](http://exchange.kw/).
participatory research partnership?.kw.
partnership [research.kw](http://research.kw/).
Patient [participation.kw](http://participation.kw/).
researcher-stakeholder [collaboration.kw](http://collaboration.kw/).
or/44-64
43 or 65
exp data collection method/
exp program evaluation/
benchmarking/
"evaluation and follow up"/
outcome assessment/
exp performance measurement system/
reproducibility/
psychometry/
((outcome? or impact?) and (measur* or assess* or evaluat*)).ab,ti.
((outcome? or impact?) and (tool? or instrument?)).ab,ti.
((measur* or assess* or evaluat*) and (tool? or instrument?)).ab,ti.
67 or 68 or 69 or 70 or 71 or 72 or 73 or 74 or 75 or 76 or 77
66 and 78

# **Appendix S4.** Health Research Partnership Pragmatic Tool Evaluation Criteria

**Health Research Partnership Tool Pragmatic Assessment: Instructions, Criteria and Assessment Grid**

**(***Modified with permission from the Patient and Public Engagement Evaluation Toolkit Project -* *Boivin et al, 2018)*

Four criteria domains are defined:

- **Scientific Rigor:** Was the development of the tool scientifically rigorous and based on existing evidence pertaining to health research partnership outcome and impact assessment?
- **Partner Perspective:** Does the tool take into account partner views in its development/modification, use, evaluation and/or validation?
- **Comprehensiveness:** Does the tool comprehensively assess the context, process, outcomes and/or impacts of health research partnerships?
- **Usability:** Is the tool easy to use?

Each criterion has 5 question items.

Score 1 point per item if the answer is YES, score 0 if the answer is NO or CANNOT ANSWER.

|  | **Question** | **Notes** |
| --- | --- | --- |
| **Scientific Rigor (SR)** | | |
| SR1 | Is the tool based on a comprehensive literature review on the assessment of outcomes and/or impacts of health research partnerships? | To answer YES, authors must cite a synthesis study (e.g., scoping, systematic, narrative review) OR report at least two electronic sources were searched, including years and databases used (e.g., Central, EMBASE, and MEDLINE). |
| SR2 | Is the evaluation tool based on the experience/expertise of key partners? | To answer YES, the paper must explicitly state that key partners (e.g., researchers, patients, clinicians, policy makers etc.) were consulted during the process of development or modification, use, evaluation and/or validation of the tool. |
| SR3 | Is the tool based on a conceptual/theoretical framework involving health research partnership outcomes and/or impact assessment? | To answer YES, the framework must include or relate to the concept of health research partnership outcomes and/or impact assessment and describe how it is operationalized. |
| SR4 | Was the tool tested for validity (i.e., the tool evaluates what it is purported to evaluate)? | To answer YES, the tool must have evidence for validity (any source). |
| SR5 | Was the tool tested for reliability (i.e., the tool produces stable and consistent results)? | To answer YES, the tool must have evidence for reliability (any source). |

| **Partner Perspective (PP)** | | |
| --- | --- | --- |
| PP1 | Were partners involved as co-designers in the development or modification, use, evaluation and/or validation of the tool? | To answer YES, partners must have been co-designers during any stage of the development/modification, use, evaluation and/or validation of the tool. |
| PP2 | Is the tool designed to be self-administered by partners? | To answer YES, relevant partners must be explicitly identified as the target users of the tool. |
| PP3 | Does the tool explicitly state that partnership outcome and impact assessment results must be reported back to partners? | To answer YES, there must be an explicit statement that results will be communicated back to partners. |
| PP4 | Was the tool specifically designed to assess level of involvement among partners in health research partnerships? | To answer YES, there must be a clear statement that the tool assesses the level of partner involvement (e.g., on the IAP2 spectrum or other level of involvement scale). |
| PP5 | Does the tool capture the influence of partners? (e.g., the extent to which partners influenced the process, final decisions, etc.) | To answer YES, the tool must ask at least one question about partners’ perceptions of partnership member’s influence. |

| **Comprehensiveness (C)** | | |
| --- | --- | --- |
| C1 | Does the tool document the context of the health research partnership? | To answer YES, the tool must ask at least one question about the internal and/or external partnership context (e.g., the nature of the issue). |
| C2 | Does the tool assess the partnership process? | To answer YES, the tool must have at least one question about the partnering process. |
| C3 | Does the tool document the outcome and/or impact of the health research partnership? | To answer YES, the tool must have at least one question about the perceived outcomes/impacts of health research partnership, including process and summative outcomes. |
| C4 | Does the tool monitor the partnering process at multiple moments? | To answer YES, the tool is explicitly designed to be used more than once during the project. |
| C5 | Does the tool consist of both open- and closed-ended questions? | To answer YES, the tool must consist of a combination of both types of questions (one of each at least). |

| **Usability (U)** | | |
| --- | --- | --- |
| U1 | Is the purpose of the tool stated? | To answer YES, the purpose of the evaluation tool must be explicitly stated. |
| U2 | Is the evaluation tool freely accessible? | To answer YES, the evaluation tool should be freely accessible through an open access journal or publicly available on the Web. |
| U3 | Is the evaluation available in an applicable format? | To answer YES, the tool should be accessible in its complete form, and be ready to use. |
| U4 | Is the evaluation tool easy to read and understand? | To answer YES, the readability score must be reported in the paper (and be in readable score range), or the tool co-designed/piloted with relevant partners prior to use |
| U5 | Is the tool accompanied by instructions for use? | To answer YES, instructions must be provided about how to use the tool in the text or in the tool itself. |

**Reporting Scores:** A total criterion score for each of the four criteria will be calculated out of 5 points, for each study. These four criteria scores will be tallied and presented graphically as a percentage out of 100.

# **Appendix S5.** Quality Assessment Checklist for Survey Studies in Psychology (Q-SSP) Criteria

(Table adapted from the Quality Assessment Checklist for Survey Studies in Psychology (Q-SSP) and Guide - Protogerou & Hagger, 2019). Each item is scored Yes/No/Not Clear/Not Applicable.

| **Item** | **Question** |
| --- | --- |
| Introduction | 1. Was the problem or phenomenon under investigation defined, described, and justified? |
| Introduction | 2. Was the population under investigation defined, described, and justified? |
| Introduction | 3. Were specific research questions and/or hypotheses stated? |
| Introduction | 4. Were operational definitions of all study variables provided? |
| Participants | 5. Were participant inclusion criteria stated? |
| Participants | 6. Was the participant recruitment strategy described? |
| Participants | 7. Was a justification/ rationale for the sample size provided? |
| Data | 8. Was the attrition rate provided? |
| Data | 9. Was a method of treating attrition provided? |
| Data | 10. Were the data analysis techniques justified (i.e., was the link between hypotheses/ aims / research questions and data analyses explained)? |
| Data | 11. Were the measures provided in the report (or in a supplement) in full? |
| Data | 12. Was evidence provided for the validity of the measure(s) or instrument(s) used? |
| Data | 13. Was information provided about the person(s) who collected the data (e.g., training, expertise, other demographic characteristics)? |
| Data | 14. Was information provided about the context (e.g., place) of data collection? |
| Data | 15. Was information provided about the duration (or start and end date) of data collection? |
| Data | 16. Was the study sample described in terms of key demographic characteristics? |
| Data | 17. Was discussion of findings confined to the population from which the sample was drawn? |
| Ethics | 18. Were participants asked to provide (informed) consent or assent? |
| ­Ethics | 19. Were participants debriefed at the end of data collection? |
| Ethics | 20. Were funding sources or conflicts of interest disclosed? |

**Scoring Rubric**

Items are scored 1 (Yes), 0 (No or Not Clear) and items scored NA are removed from the denominator. The overall quality score (%) is a percentage calculated by dividing Yes (Y) scores by the Total (T) number of applicable items multiplied by 100. Reports failing to attain a Y score for 5 items are categorized ‘questionable quality’.

For denominators:

If (T) = 20, a score of Y/T ≥ 75% is ‘acceptable quality’.

If Y/T <75%, the study is of ‘questionable quality’.

**Domain Scores**

Domain scores are presented as a simple ratio of the (Y) items, divided by the (T) applicable items.

1. Introduction Score (4 items – Rationale, Variables): Y/4
2. Participants Score (3 items – Sampling, Recruitment): Y/3
3. Data Score (10 items – Data collection, analyses, measures, results, discussion): Y/10
4. Ethics Score (3 items): Y/3

# **Appendix S6:** Bibliography of included studies

| **Eligible Papers (n=48 studies, 1 companion report; n=58 tools)** |
| --- |
| 1. Butterfoss FD, Goodman RM, Wandersman A. Community coalitions for prevention and health promotion: factors predicting satisfaction, participation, and planning. Health Education Quarterly. 1996;23(1):65-79. 2. Kegler MC, Steckler A, McLeroy K, Malek SH. Factors that contribute to effective community health promotion coalitions: a study of 10 Project ASSIST coalitions in North Carolina. Health Education & Behavior. 1998;25(3):338-353. 3. Chan B, Bazzoli G, Shortell SM, Hasnain-Wynia R. A social capital index for community partnerships. International Quarterly of Community Health Education. 2000;20(3):213-235. 4. Shortell SM, Zukoski AP, Alexander JA, et al. Evaluating partnerships for community health improvement: tracking the footprints. J Health Politics, Policy & Law. 2002;27(1):49-91. 5. Weiss ES, Anderson RM, Lasker RD. Making the most of collaboration: exploring the relationship between partnership synergy and partnership functioning. Health Education & Behavior. 2002;29(6):683-698. 6. El Ansari W, Phillips CJ. The costs and benefits to participants in community partnerships: a paradox? Health Promotion Practice. 2004;5(1):35-48. 7. El Ansari W, Phillips CJ, Zwi AB. Public health nurses' perspectives on collaborative partnerships in South Africa. Public Health Nursing. 2004;21(3):277-286. 8. Metzger ME, Alexander JA, Weiner BJ. The effects of leadership and governance processes on member participation in community health coalitions. Health Education & Behavior. 2005;32(4):455-473. 9. Kegler MC, Williams CW, Cassell CM, et al. Mobilizing communities for teen pregnancy prevention: associations between coalition characteristics and perceived accomplishments. Journal of Adolescent Health. 2005;37(3 Suppl):S31-41. 10. Cramer ME, Atwood JR, Stoner JA. Measuring community coalition effectiveness using the ICE instrument. Public Health Nursing. 2006;23(1):74-87. 11. Feinberg ME, Bontempo DE, Greenberg MT. Predictors and level of sustainability of community prevention coalitions. American Journal of Preventive Medicine. 2008;34(6):495-501. 12. Feinberg ME, Gomez BJ, Puddy RW, Greenberg MT. Evaluation and community prevention coalitions: validation of an integrated Web-based/technical assistance consultant model. Health Education & Behavior. 2008;35(1):9-21. 13. Orr Brawer CR. Replication of the value template process in a community coalition: Implications for social capital and sustainability, ProQuest Information & Learning; 2008. 14. King G, Servais M, Kertoy M, et al. A measure of community members' perceptions of the impacts of research partnerships in health and social services. Evaluation & Program Planning. 2009;32(3):289-299. 15. Tolma EL, Cheney MK, Troup P, Hann N. Designing the process evaluation for the collaborative planning of a local turning point partnership. Health Promotion Practice. 2009;10(4):537-548. 16. Wagemakers A, Koelen MA, Lezwijn J, Vaandrager L. Coordinated action checklist: a tool for partnerships to facilitate and evaluate community health promotion. Global Health Promotion. 2010;17(3):17-28. 17. King G, Servais M, Forchuk C, et al. Features and impacts of five multidisciplinary community-university research partnerships. Health & Social Care in the Community. 2010;18(1):59-69. 18. Ziff MA, Willard N, Harper G. Connect to Protect Researcher Community Partnerships: Assessing Change in Successful Collaboration Factors over Time. Global Journal of Community Psychology Practice, 2010, 1(1): 32-39. 19. Jones J, Barry MM. Developing a scale to measure synergy in health promotion partnerships. Global Health Promotion. 2011;18(2):36-44. 20. Jones J, Barry M. Developing a scale to measure trust in health promotion partnerships. Health Promot Int. 2011 Dec;26(4):484-91. doi: 10.1093/heapro/dar007. Epub 2011 Feb 20. 21. Perkins DF, Feinberg ME, Greenberg MT, et al. Team factors that predict to sustainability indicators for community-based prevention teams. Evaluation & Program Planning. 2011;34(3):283-291. 22. Bilodeau A, Galarneau M, Fournier M, et al. L'Outil diagnostique de l'action en partenariat: fondements, élaboration et validation. Canadian Journal of Public Health. 2011;102(4):298-302.   English translation companion report:  Bilodeau A, Kranias G. Self-Evaluation Tool for Action in Partnership: Translation and Cultural Adaptation of the Original Quebec French Tool to Canadian English. Canadian Journal of Program Evaluation, 2019, 34(2): 192-206 doi 10.3138/cjpe.43685   1. Curro FA, Thompson VP, Grill A, et al. An assessment of the perceived benefits and challenges of participating in a practice-based research network. Primary Dental Journal. 2012;1(1):50-57. 2. El Ansari W. Leadership in community partnerships: South African study and experience. Central European Journal of Public Health. 2012;20(3):174-184. 3. Brown LD, Feinberg ME, Greenberg MT. Measuring Coalition Functioning: Refining Constructs through Factor Analysis. Health Educ Behav. 2012 August ; 39(4): 486–497. doi:10.1177/1090198111419655. 4. Nargiso JE, Friend KB, Egan C, et al. Coalitional capacities and environmental strategies to prevent underage drinking. American Journal of Community Psychology. 2013;51(1-2):222-231. 5. Perkins C-TM. Partnership functioning and sustainability in nursing academic practice partnerships: The mediating role of partnership synergy, University of Northern Colorado; 2014. 6. Chang FC, Liu CH, Liao LL, et al. Facilitating the implementation and efficacy of health-promoting schools via an action-research approach in Taiwan. Health Promotion International. 2014;29(2):306-316. 7. Brown LD, Feinberg ME, Shapiro VB, Greenberg MT. Reciprocal relations between coalition functioning and the provision of implementation support. Prevention Science. 2015;16(1):101-109. 8. Bornstein DB, Pate RR, Beets MW, Ortaglia A, Saunders RP, Blair SN. New Perspective on Factors Related to Coalition Success: Novel Findings From an Investigation of Physical Activity Coalitions Across the United States. Journal of Public Health Management & Practice. 2015;21(6):E23-30. 9. Oetzel JG, Villegas M, Zenone H, White Hat ER, Wallerstein N, Duran B. Enhancing stewardship of community-engaged research through governance. American Journal of Public Health. 2015;105(6):1161-1167. 10. Oetzel JG, Zhou C, Duran B, et al. Establishing the psychometric properties of constructs in a community-based participatory research conceptual model. American Journal of Health Promotion. 2015;29(5):e188-202. 11. Stocks SJ, Giles SJ, Cheraghi-Sohi S, Campbell SM. Application of a tool for the evaluation of public and patient involvement in research. BMJ Open. 2015;5(3):e006390. 12. Brown LD, Chilenski SM, Ramos R, Gallegos N, Feinberg ME. Community Prevention Coalition Context and Capacity Assessment: Comparing the United States and Mexico. Health Education & Behavior. 2016;43(2):145-155. 13. Goodman MS, Sanders Thompson VL, Johnson CA, et al. Evaluating Community Engagement in Research: Quantitative Measure Development. Journal of Community Psychology. 2017;45(1):17-32. 14. Okazaki S, Wong SN, Kaplan BL. Strategic collaborative partnerships to improve immigrant Chinese community health: A case study. Asian American Journal of Psychology. 2017;8(4):339-350. 15. Jones J, Barry MM. Factors influencing trust and mistrust in health promotion partnerships. Global Health Promotion. 2018;25(2):16-24. 16. West KM. Researcher trustworthiness in community-academic research partnerships: Implications for genomic research, ProQuest Information & Learning; 2018. 17. Oetzel JG, Wallerstein N, Duran B, et al. Impact of Participatory Health Research: A Test of the Community-Based Participatory Research Conceptual Model. BioMed Research International. 2018; 7281405. 18. Duran, B., et al. (2019). "Toward Health Equity: A National Study of Promising Practices in Community-Based Participatory Research." Progress in Community Health Partnerships 13(4): 337-352. 19. Soobiah, C., et al. (2019). "Engaging knowledge users in a systematic review on the comparative effectiveness of geriatrician-led models of care is possible: A cross-sectional survey using the Patient Engagement Evaluation Tool." Journal of Clinical Epidemiology 113: 58-63. 20. Dickson, E., et al. (2020). "Characteristics and Practices Within Research Partnerships for Health and Social Equity." Nursing Research 69(1): 51-61. 21. Rodríguez Espinosa, P., et al. (2020). "Personal Outcomes in Community‐based Participatory Research Partnerships: A Cross‐site Mixed Methods Study." American Journal of Community Psychology 66(3/4): 439-449. 22. Lucero, J. E., et al. (2020). "Engage for Equity: The Role of Trust and Synergy in Community-Based Participatory Research." Health Education & Behavior 47(3): 372-379. 23. van Schelven, F., et al. (2021). "Exploring the impact of patient and public involvement with young people with a chronic condition: A multilevel analysis." Child: Care, Health and Development 47(3): 349-356. 24. Hamilton, C. B., et al. (2021). "Shortening and validation of the patient engagement in research scale (peirs) for measuring meaningful patient and family caregiver engagement." Health Expectations: An International Journal of Public Participation in Health Care & Health Policy. 25. Boursaw, B., et al. (2021). "Scales of practices and outcomes for community‐engaged research." American Journal of Community Psychology. 26. Loban, E., et al. (2021). "Measuring partnership synergy and functioning: Multi-stakeholder collaboration in primary health care." PLoS ONE 16(5 May): e0252299. |

# **Appendix S7.** PRISMA-Systematic Review Checklist

| **Section and Topic** | **Item #** | **Checklist item** | **Location where item is reported** |
| --- | --- | --- | --- |
| **TITLE** | | |  |
| Title | 1 | Identify the report as a systematic review. | P1 |
| **ABSTRACT** | | |  |
| Abstract | 2 | See the PRISMA 2020 for Abstracts checklist. | P4-5 |
| **INTRODUCTION** | | |  |
| Rationale | 3 | Describe the rationale for the review in the context of existing knowledge. | P6-7 |
| Objectives | 4 | Provide an explicit statement of the objective(s) or question(s) the review addresses. | P7-8 |

| **METHODS** | | |  |
| --- | --- | --- | --- |
| Eligibility criteria | 5 | Specify the inclusion and exclusion criteria for the review and how studies were grouped for the syntheses. | P9 |
| Information sources | 6 | Specify all databases, registers, websites, organisations, reference lists and other sources searched or consulted to identify studies. Specify the date when each source was last searched or consulted. | P9 |
| Search strategy | 7 | Present the full search strategies for all databases, registers and websites, including any filters and limits used. | Supplement File 1 (Appendix 3, p6-13) |
| Selection process | 8 | Specify the methods used to decide whether a study met the inclusion criteria of the review, including how many reviewers screened each record and each report retrieved, whether they worked independently, and if applicable, details of automation tools used in the process. | P9-12 |
| Data collection process | 9 | Specify the methods used to collect data from reports, including how many reviewers collected data from each report, whether they worked independently, any processes for obtaining or confirming data from study investigators, and if applicable, details of automation tools used in the process. | P9-12 |
| Data items | 10a | List and define all outcomes for which data were sought. Specify whether all results that were compatible with each outcome domain in each study were sought (e.g. for all measures, time points, analyses), and if not, the methods used to decide which results to collect. | P9-12, Supplement File 1 (Appendix 1, p2-3) |
|  | 10b | List and define all other variables for which data were sought (e.g. participant and intervention characteristics, funding sources). Describe any assumptions made about any missing or unclear information. | P9-12, Supplement File 1 (Appendix 1, p2-3) |
| Study risk of bias assessment | 11 | Specify the methods used to assess risk of bias in the included studies, including details of the tool(s) used, how many reviewers assessed each study and whether they worked independently, and if applicable, details of automation tools used in the process. | P11-12 |
| Effect measures | 12 | Specify for each outcome the effect measure(s) (e.g. risk ratio, mean difference) used in the synthesis or presentation of results. | NA |
| Synthesis methods | 13a | Describe the processes used to decide which studies were eligible for each synthesis (e.g. tabulating the study intervention characteristics and comparing against the planned groups for each synthesis (item #5)). | P11 |
|  | 13b | Describe any methods required to prepare the data for presentation or synthesis, such as handling of missing summary statistics, or data conversions. | NA |
|  | 13c | Describe any methods used to tabulate or visually display results of individual studies and syntheses. | NA |
|  | 13d | Describe any methods used to synthesize results and provide a rationale for the choice(s). If meta-analysis was performed, describe the model(s), method(s) to identify the presence and extent of statistical heterogeneity, and software package(s) used. | NA |
|  | 13e | Describe any methods used to explore possible causes of heterogeneity among study results (e.g. subgroup analysis, meta-regression). | NA |
|  | 13f | Describe any sensitivity analyses conducted to assess robustness of the synthesized results. | NA |
| Reporting bias assessment | 14 | Describe any methods used to assess risk of bias due to missing results in a synthesis (arising from reporting biases). | NA |
| Certainty assessment | 15 | Describe any methods used to assess certainty (or confidence) in the body of evidence for an outcome. | NA |
| **RESULTS** | | |  |
| Study selection | 16a | Describe the results of the search and selection process, from the number of records identified in the search to the number of studies included in the review, ideally using a flow diagram. | P12-18, Fig 1 |
|  | 16b | Cite studies that might appear to meet the inclusion criteria, but which were excluded, and explain why they were excluded. | NA |
| Study characteristics | 17 | Cite each included study and present its characteristics. | P12-18, Tables 1-7, Figures 1-4 |
| Risk of bias in studies | 18 | Present assessments of risk of bias for each included study. | P17, Table 7 |
| Results of individual studies | 19 | For all outcomes, present, for each study: (a) summary statistics for each group (where appropriate) and (b) an effect estimate and its precision (e.g. confidence/credible interval), ideally using structured tables or plots. | NA |
| Results of syntheses | 20a | For each synthesis, briefly summarise the characteristics and risk of bias among contributing studies. | P12-18, Tables 1-7, Figures 1-4 |
|  | 20b | Present results of all statistical syntheses conducted. If meta-analysis was done, present for each the summary estimate and its precision (e.g. confidence/credible interval) and measures of statistical heterogeneity. If comparing groups, describe the direction of the effect. | NA |
|  | 20c | Present results of all investigations of possible causes of heterogeneity among study results. | NA |
|  | 20d | Present results of all sensitivity analyses conducted to assess the robustness of the synthesized results. | NA |
| Reporting biases | 21 | Present assessments of risk of bias due to missing results (arising from reporting biases) for each synthesis assessed. | NA |
| Certainty of evidence | 22 | Present assessments of certainty (or confidence) in the body of evidence for each outcome assessed. | NA |
| **DISCUSSION** | | |  |
| Discussion | 23a | Provide a general interpretation of the results in the context of other evidence. | P18-24 |
|  | 23b | Discuss any limitations of the evidence included in the review. | P21-24 |
|  | 23c | Discuss any limitations of the review processes used. | P21-24 |
|  | 23d | Discuss implications of the results for practice, policy, and future research. | P23-24 |

| **OTHER INFORMATION** | | |  |
| --- | --- | --- | --- |
| Registration and protocol | 24a | Provide registration information for the review, including register name and registration number, or state that the review was not registered. | P5, P8 |
|  | 24b | Indicate where the review protocol can be accessed, or state that a protocol was not prepared. | P5, P8 |
|  | 24c | Describe and explain any amendments to information provided at registration or in the protocol. | P8, Supplement File 1 (Appendix 1, P2-3) |
| Support | 25 | Describe sources of financial or non-financial support for the review, and the role of the funders or sponsors in the review. | P25 |
| Competing interests | 26 | Declare any competing interests of review authors. | P24-25 |
| Availability of data, code and other materials | 27 | Report which of the following are publicly available and where they can be found: template data collection forms; data extracted from included studies; data used for all analyses; analytic code; any other materials used in the review. | P24 |

*From:*  Page MJ, McKenzie JE, Bossuyt PM, Boutron I, Hoffmann TC, Mulrow CD, et al. The PRISMA 2020 statement: an updated guideline for reporting systematic reviews. BMJ 2021;372:n71. doi: 10.1136/bmj.n71

# **References**

1. Sampson, M., McGowan, J., Cogo, E., Grimshaw, J., Moher, D., Lefebvre, C., *An evidence-based practice guideline for the peer review of electronic search strategies.* Journal of Clinical Epidemiology, 2009. **62**: p. 944-952.

2. McGowan, J., Sampson, M., Salzwedel, D., Cogo, E., Foerster, V., Lefebvre, C., *Guideline Statement: PRESS Peer Review of Electronic Search Strategies 2015 Guideline Statement.* Journal of Clinical Epidemiology, 2016. **75**: p. 40-46.

3. Porter, L., *Planning in (post) colonial settings: Challenges for theory and practice.* Planning Theory and Practice, 2006. **7**(4): p. 383-396.

4. Hoekstra, F., Mrklas, K.J.*, Sibley, K., Nguyen, T., Vis-Dunbar, M., Neilson, C.J., Crockett, L.K., Gainsforth, H.L.^, Graham, I.D^. (*co-authors, ^co-senior authors), *A Review Protocol on Research Partnerships: A Coordinated Multicenter Team Approach.* Systematic Reviews, 2018. **7**(217): p. 1-14.

5. University of Waterloo. *Research Ethics: Definition of a health outcome.* 2018 [cited 2018 March 7]; Available from: <https://uwaterloo.ca/research/office-research-ethics/research-human-participants/pre-submission-and-training/human-research-guidelines-and-policies-alphabetical-list/definition-health-outcome>.

6. Higher Education Funding Council for England: Research Excellence Framework 2014. *Assessment framework and guidance on submissions 2011.* 2014 [cited 14 Nov 2017; Available from: <http://www.ref.ac.uk/2014/media/ref/content/pub/assessmentframeworkandguidanceonsubmissions/GOS%20including%20addendum.pdf>.

7. Stanick, C.F., Halko, H.M., Nolen, E.A., Powell, B.J., Dorsey, C.N., Mettert, K.D, Weiner, B.J., Barwick, M., Wolfenden, L., Damschroder, L.J., Lewis, C.C., *Pragmatic measures for implementation research: development of the Psychometric and Pragmatic Evidence Rating Scale (PAPERS).* Translational Behavioural Medicine, 2021. **11**(1): p. 11-20.

8. Lewis, C.C., Mettert, K.D., Stanick, C.F., Halko, H.M., Nolen, E.A., Powell, B.J., Weiner, B.J., *The psychometric and pragmatic evidence rating scale (PAPERS) for measure development and evaluation.* Implementation Research and Practice, 2021(January).

9. Graham, I.D., Beardall, S., Carter, A.O., Glennie, J., Hebert, P.C., Tetroe, J.M., McAlister, F.A., Visentin, S., Anderson, G.M., *What is the quality of drug therapy clinical practice guidelines in Canada?* Canadian Medical Association Journal, 2001. **165**(2): p. 157-163.

10. Centre of Excellence on Partnership with Patients and the Public (CEPPP). *Patient and Public Engagement Evaluation Toolkit*. 2021 [cited 2021 23 Nov]; Available from: <https://ceppp.ca/en/evaluation-toolkit/#care>||div1|category_evaluation-toolkit-project|1.

11. Boivin, A., L’Esperance, A., Gauvin, F.P., Dumez, V., Maccaulay, A.C., Lehoux, P., Abelson, J., *Patient and public engagement in research and health system decision making: A systematic review of evaluation tools.* Health Expectations, 2018. **21**(6): p. 1075-1084.

12. Drahota, A., Meza, R.D., Brikho, B., Naaf, M., Estabillo, J.A., Gomez, E.D., Vejnoska, S. F., Dufek, S., Stahmer, A.C., Aarons, G.A., *Community-Academic Partnerships: A systematic review of the state of the literature and recommendations for future research.* Milbank Quarterly, 2016. **94**(1): p. 163-214.

13. Mrklas, K.J., *Towards the development of a valid, reliable and acceptable tool for assessing the impact of health research partnerships (PhD dissertation thesis proposal).* 2018, University of Calgary: Calgary, Canada. p. 119pp.

14. May, C., Finch, T., Mair, F., Ballini, L., Dowrick, C., Eccles, M., Gask, L., MacFarlane, A., Murray, E., Rapley, T., Rogers, A., Treweek, S., Wallace, P., Anderson, G., Burns, J., Heaven, B., *Understanding the implementation of complex interventions in health care: the normalization process model.* BMC Health Services Research, 2007. **7**(148): p. 1-7.

15. Terwee, C.B., Bot, S., de Boer, M.R., van der Windt, D., Knol, D.L., Dekker, J., Bouter, L.M., de Vet, H.C.W.,, *Quality criteria were proposed for measurement properties of health status questionnaires.* Journal of Clinical Epidemiology, 2007. **60**: p. 34-42.

16. Bhattacherjee, A. *Social Science Research: Principles, Methods and Practices.* Textbooks Collection 3 2012 [cited 2022 11 January]; Available from: <https://digitalcommons.usf.edu/oa_textbooks/3>.

17. Kimel, M., Revicki, D.,, *Inter-rater Reliability*, in *Encyclopedia of Quality of Life and Well- Being Research.*, A.C. Michalos, Eds,. Editor. 2014, Springer: Dordrecht.

18. Davidson, M., *Known-Groups Validity*, in *Encyclopedia of Quality of Life and Well-Being Research.* 2014, Springer: Dordrecht.

19. Piedmont, R.L., *Factorial Validity*, in *Encyclopedia of Quality of Life and Well-Being Research*, A.C. Michalos, Eds,. Editor. 2014, Springer: Dordrecht.

20. Boateng, G.O., Neilands, T.B., Frongillo, E.A., Melgar-Quinonez, H.R., Young, S.L.,, *Best practices for developing and validating scales for health, social, and behavioural research.* Frontiers in Public Health, 2018. **6**(149).

21. Chien, C.C., & Yao, G.,, *Norms*, in *Encyclopedia of Quality of Life and Well-Being Research*, A.C. Michalos, Eds,. Editor. 2014, Springer: Dordrecht.
